# Respiratory microbiota resistance and resilience to pulmonary exacerbation and subsequent antimicrobial intervention

**DOI:** 10.1038/ismej.2015.198

**Published:** 2015-11-10

**Authors:** Leah Cuthbertson, Geraint B Rogers, Alan W Walker, Anna Oliver, Laura E Green, Thomas W V Daniels, Mary P Carroll, Julian Parkhill, Kenneth D Bruce, Christopher J van der Gast

**Affiliations:** 1 NERC Centre for Ecology & Hydrology, Wallingford, UK; 2 Institute of Pharmaceutical Science, Molecular Microbiology Research Laboratory, King‘s College London, London SE1 9NH, UK; 3 SAHMRI Infection and Immunity Theme, School of Medicine, Flinders University, Adelaide, South Australia, Australia; 4 Pathogen Genomics Group, Wellcome Trust Sanger Institute, Hinxton, Cambridge, UK; 5 Microbiology Group, Rowett Institute of Nutrition and Health, University of Aberdeen, Aberdeen, UK; 6 School of Life Sciences, University of Warwick, Coventry, UK; 7 Cystic Fibrosis Unit, Southampton University Hospitals NHS Trust, Southampton, UK

## Abstract

Pulmonary symptoms in cystic fibrosis (CF) begin in early life with chronic lung infections and concomitant airway inflammation leading to progressive loss of lung function. Gradual pulmonary function decline is interspersed with periods of acute worsening of respiratory symptoms known as CF pulmonary exacerbations (CFPEs). Cumulatively, CFPEs are associated with more rapid disease progression. In this study multiple sputum samples were collected from adult CF patients over the course of CFPEs to better understand how changes in microbiota are associated with CFPE onset and management. Data were divided into five clinical periods: pre-CFPE baseline, CFPE, antibiotic treatment, recovery, and post-CFPE baseline. Samples were treated with propidium monoazide prior to DNA extraction, to remove the impact of bacterial cell death artefacts following antibiotic treatment, and then characterised by 16S rRNA gene-targeted high-throughput sequencing. Partitioning CF microbiota into core and rare groups revealed compositional resistance to CFPE and resilience to antibiotics interventions. Mixed effects modelling of core microbiota members revealed no significant negative impact on the relative abundance of *Pseudomonas aeruginosa* across the exacerbation cycle. Our findings have implications for current CFPE management strategies, supporting reassessment of existing antimicrobial treatment regimens, as antimicrobial resistance by pathogens and other members of the microbiota may be significant contributing factors.

## Introduction

Cystic fibrosis (CF) is a common recessive genetic disorder, primarily of Caucasians, affecting more than 8000 children and adults in the UK and an estimated 100 000 globally (Davies *et al.*, 2014). Although this disorder is multisystemic, the leading cause of morbidity and mortality in an overwhelming majority (*ca*. 90%) of patients is chronic lung infections and concomitant airway inflammation (Foweraker, 2009). Until a treatment for the disorder can be found, for example, cystic fibrosis transmembrane conductance regulator gene therapy or therapies that improve cystic fibrosis transmembrane conductance regulator function, the best way to continue to raise life expectancy is to improve treatment of the chronic polymicrobial lung infections (LiPuma, 2014). Implicit within the aim of improving and directing treatment is to better understand the nature of those chronic polymicrobial infections, their response to standard treatments, and how they differ and change with disease progression.

Pulmonary symptoms in CF begin in early life and over time a combination of impaired mucociliary clearance, innate immune responses, and chronic infection lead to a progressive loss of lung function (Flume *et al.*, 2009). This gradual decline in pulmonary function is interspersed with periods of acute worsening of respiratory symptoms known as CF pulmonary exacerbations (CFPEs) (Sibley *et al.*, 2008). Cumulatively, these periods of CFPE are associated with more rapid disease progression and reduced survival as well as a recognised reduction in quality of life and an increase in overall healthcare costs (VanDevanter *et al.*, 2013). There is currently no consensus for a generally applicable definition to describe when patients are experiencing a CFPE. This is mainly due to a lack of consistency in the symptoms experienced by CF patients of different ages and disease state, thus making it challenging for paediatric and adult physicians to come to an agreement (Smyth and Elborn, 2008). Despite this, clinical features can include decreased exercise tolerance, increased cough and sputum production, shortness of breath, chest pain, absence from school or work, increased adventitial sounds on lung examination, decreased appetite or weight loss, and a decline in lung function (Fuchs *et al.*, 1994). Although lung function is still widely relied upon as an indicator of worsening symptoms, it has been revealed that short-term changes in lung function show little effect on the sensitivity or specificity of CFPE diagnosis (Rosenfeld *et al.*, 2001).

As such, clinicians face the challenging role of determining if a patient is experiencing a CFPE and hence when intervention is appropriate. CFPEs are treated with high-dose intravenous antibiotics that are broad spectrum in their activity, but expected to have an impact on *Pseudomonas aeruginosa* (Smyth and Elborn, 2008). It is now recognised that immediate management of CFPE results in improved prognosis and increased likelihood of patient recovery, underlining the importance of CFPE diagnosis (Sanders *et al.*, 2010). Therefore, there is an urgent need to identify and develop more reliable bioindicators of disease state change.

Studies using ecological analyses to aid in the interpretation of culture, independent data have shown associations between respiratory microbiota characteristics and clinical outcomes (LiPuma, 2014). For example, increasing age, disease progression, and reduced lung function have been associated with related changes in CF lung microbiota, summarised in reduced bacterial diversity and increasingly conserved community composition (e.g. Cox *et al.*, 2010; Klepac-Ceraj *et al.*, 2010; van der Gast *et al.*, 2011; Zhao *et al.*, 2012; van der Gast *et al.*, 2014). However, a fundamental knowledge gap remains in relation to how the CF microbiota behaves and respond to CFPE and subsequent antibiotic interventions, largely due to a paucity of studies on this subject.

A useful framework proposed to better understand changes in microbiota associated with CFPE and antibiotic intervention has been to partition data into defined clinical periods: (B) baseline, (E) CFPE, (T) treatment, and (R) recovery (Zhao *et al.*, 2012; Price *et al.*, 2013). Set within this BETR classification, studies have provided useful insights including a clear demonstration that CFPE do not result from increased total bacterial density or density of a specific recognised CF pathogen, such as *P. aeruginosa* (Stressmann *et al.*, 2011; Fodor *et al.*, 2012; Carmody *et al.*, 2013; Price *et al.*, 2013). Conversely, how changes in microbiota composition and structure are associated with CFPE and intervention remain less clear; as different studies have shown either significant change or no change in CF bacterial communities, and in specific constituent species (Fodor *et al.*, 2012; Stressmann *et al.*, 2012; Daniels *et al.*, 2013; Price *et al.*, 2013; Smith *et al.*, 2014). These contradictory findings may be attributed to differing experimental designs, patient cohorts, and sampling strategies. For example, studies have typically relied on single-point samples to be representative of a given disease state (e.g. baseline or exacerbation) which will not account for variance in either microbiota or clinical factors, such as lung function (commonly, FEV_1_, forced expiratory volume in 1 s), in a given clinical period (Carmody *et al.*, 2013; Price *et al.*, 2013; Zemanick *et al.*, 2013). Studies have also been based in the main on point samples from subsets and different combinations of the clinical periods defined above (Fodor *et al.*, 2012; Carmody *et al.*, 2013; Price *et al.*, 2013; Smith *et al.*, 2014), making them difficult to compare, and as such the complete cycle of clinical periods from pre- to post-CFPE baseline, that is BETRB, has not been studied. However, in both instances this may be a reflection of the difficulty in obtaining respiratory samples and metadata over this type of longitudinal period.

In the current study, multiple sputum samples were collected from 10 CF adult patients over the course of a CFPE. The data were partitioned into five clinical periods: (B_0_) baseline pre-CFPE; (E) CFPE, 30 days prior to treatment; (T) treatment for CFPE; (R) recovery, 30 days post-CFPE; and (B_1_) baseline post-CFPE. In addition, multiple sputum samples were collected from two adult CF patients who were clinically stable over the same time period (B_0_ only). During periods B_0_, E, R, and B_1_, patients were receiving their standard doses of maintenance antibiotics. For T, patients were hospitalised and receiving increased antibiotic intervention, which was typically intravenous antibiotics (Table 1). To allow profiling of the extant CF lung microbiota, sputum samples were treated with propidium monoazide prior to DNA extraction, to remove the impact of extracellular DNA or DNA from dead or damaged bacterial cells, and then 16S rRNA gene-targeted high-throughput sequencing was carried out (Rogers *et al.*, 2013). This allowed a detailed examination of (1) how changes in CF microbiota are associated with the onset of CFPEs, (2) the effect of antibiotic therapy administered for treatment of CFPE on the CF microbiota, and (3) identification of potential biomarkers of disease state change.

## Materials and methods

### Patients and clinical samples

This study was undertaken with the local ethical approval from Southampton and South West Hampshire Research Ethics Committee (06/Q1704/24). Sputum samples were collected from 12 adult CF patients attending the Southampton General Hospital, Southampton, UK (Table 1). All sputum samples were stored at 4^o^C immediately after expectoration and transferred to −80 °C storage within 12 h of expectoration. This timeframe was selected as recent evidence suggests that failure to stabilise samples at −80 °C within 12 h significantly impacts sequence-based microbiota profiling. (Cuthbertson *et al.*, 2014). Subjects were selected due to their persistent production of sputum and history of CFPE. All patients were clinically defined as chronically colonised with *P. aeruginosa*. Samples were collected from patients during periods of baseline, defined as periods where patients were only receiving maintenance doses of antibiotics. During the study period 10 of the 12 patients were treated for a CFPE. The start and end of CFPE were identified by treating clinicians, and were defined for the purpose of this study as the period of time where patients received clinical intervention in the form of antibiotic treatment. The antibiotics administered to each patient are all broad spectrum in activity and are expected to have an impact on the predominant pathogen, *P. aeruginosa* (Table 1). Decisions to initiate treatment were based on worsening clinical symptoms (Fuchs *et al.*, 1994). Stabilisation or improvement of these symptoms led to the termination of this treatment. Retrospectively samples were partitioned into five periods: (B_0_) baseline pre-CFPE; (E) CFPE, within 30 days prior to antibiotic treatment; (T) period of time patients were receiving treatment for pulmonary exacerbation; (R) recovery, within 30 days post-treatment for CFPE; and (B_1_) baseline post-CFPE. Clinically relevant symptoms were monitored throughout the sampling period. Lung function (FEV_1_) was assessed using a Koko PeakPro home spirometer (Ferraris Cardiorespiratory, Louisville, CO, USA) at the time of each sample collection. Patients were also required to assess respiratory symptoms: cough, breathlessness, sputum production, and general wellbeing, using a visual analogue scale scored 0–100, with 0 being the worst and 100 the best.

### DNA extraction and pyrosequencing

Sputum samples were washed three times with 1 × phosphate-buffered saline to remove saliva as previously described (Rogers *et al.*, 2006). Extracellular DNA and DNA from nonviable cells were excluded from analysis via cross-linking with propidium monoazide prior to DNA extraction, as previously described (Rogers *et al.*, 2013). Barcode encoded FLX amplicon pyrosequencing was performed using the primer 338F (5′-ACTCCTACGGGAGGCAGCAG) and 926R (5′-CCGTCAATTCMTTTRAGT) as described previously (Cooper *et al.*, 2013). Except, initial generation of 16S rRNA gene amplicons involved a one-step polymerase chain reaction of 25 cycles using *AccuPrime Taq* DNA Polymerase High Fidelity (Invitrogen, Carlsbad, CA, USA). polymerase chain reaction-negative controls were included in each sequencing run (Salter *et al.*, 2014). Four hundred and fifty-four pyrosequencing using the Lib-L kit was performed at the Wellcome Trust Sanger Institute (Hinxton, UK).

### Sequence analysis

The mothur sequencing analysis platform was used to analyse the resulting data (Schloss *et al.*, 2011). Sequence quality was ensured by trimming reads where the average quality score fell below 35 across a rolling window of 50 bases. Reads shorter than 400 bp in length and those that included mismatches in the barcode or 16S rRNA gene primer sequence, ambiguous base calls and homopolymeric stretches longer than 8 bases were subsequently removed. Chimeras were removed in mothur using the Perseus software program (Quince *et al.*, 2011). Sequences were assembled into operational taxonomic units (OTUs) at 97% sequence identity to give an approximation of species (Schloss and Handelsman, 2006) and identified using the Ribosomal Database Project reference database provided in mothur. OTUs identified in negative controls were removed from further analysis (Salter *et al.*, 2014).

As the Ribosomal Database Project classifier only provides classifications down to the genus level, representative sequences from each OTU were used to give appropriate species-level identifications using MegaBlast against the NCBI nucleotide archive. For some OTUs multiple, equally scoring, matches were obtained, As such, species-level designations should be considered putative. The raw sequence data reported in this study have been deposited in the European Nucleotide Archive under study accession numbers ERP005251 and ERP007059, and sample accession numbers ERS421603 and ERS551400. The relevant barcode information for each sample is shown in Supplementary Table S1.

### Statistical analysis

Species were partitioned into core and rare species groups using a modification of the method previously described (van der Gast *et al.*, 2011). Based on a significant positive distribution–abundance relationship, the persistent and abundant core species were defined as those in more than 75% of all samples, while all other species falling outside of the upper quartile were considered to be rare (Hedin *et al.*, 2015). Species turnover between consecutive samples was measured using the method described by Brown and Kodric-Brown (1977). Turnover was defined as *t*=*b*+*c*/*S*_1_+*S*_2_. Where *b*=the number of species present only in the first sample; *c*=the number of species present only in the second sample; *S*_1_=the total number of species in the first sample; and *S*_2_ the total number of species in the second sample (Brown and Kodric-Brown, 1977). Species richness (S*) was used as previously described (Rogers *et al.*, 2013). In brief, S* was calculated with a uniform re-sample size (to match the smallest sequence size in each group) following 1000 iterations in each instance and performed in R version 3.1.1 (Oksanen *et al.*, 2013; The R Development Core Team, 2013). Two sample *t*-tests, regression analysis, coefficients of determination (*r*^2^), residuals and significance (*P*) were calculated using Minitab software (version 16; Minitab, University Park, PA, UK). Analysis of similarity (ANOSIM) and similarity of percentages analysis (SIMPER) were performed using the PAST (Paleontological Statistics, version 3.01) program available from the University of Oslo (http://folk.uio.no/ohammer/past). The Bray–Curtis quantitative index of similarity was used as the underpinning community similarity measure for both ANOSIM and SIMPER analyses.

Mixed-effect models were fitted in R version 3.1.1 using the GLMMADMB package version 0.8.0 (The R Development Core Team, 2013). Values of *r*^*2*^ were calculated using the R package MuMIN. To analyse species-level changes over the disease periods, mixed-effect models (GLMMADMB) with negative bionomial errors were used as data were over dispersed (greater variability than would be expected based on Poisson distribution). For each species, the change in abundance across all patients was measured using the disease period as the fixed effect and variation between patients was accounted for by including patient as a random effect. The model fits changes in abundance on the logit scale. The null hypothesis for each species was there would be no change in species abundance between periods.

## Results

An expectation for patterns of lung function (FEV_1_) within individual patients was that it would entail a consistent decline in FEV_1_ from baseline (B_0_) through the CFPE and into the treatment periods, followed by an improvement in the subsequent periods. However, no common pattern in lung function by clinical period was observed across the 10 patients that experienced CFPE (Supplementary Figure S1). Therefore, within the current study, FEV_1_ was found to be a poor indicator of short-term disease state.

Microbiota diversity and composition from respiratory samples was assessed using 16S rRNA gene-targeted pyrosequencing. From 237 samples, a total of 386 002 bacterial sequence reads (mean±standard deviation (s.d.) per sample, 1628±84) were included in the final analysis, identifying 103 genera and 183 distinct OTUs classified to the species level (Supplementary Table S2); however, given the relative length of the ribosomal sequences analysed, these identities should be considered putative. The average numbers of bacterial sequence reads per sample were similar among the five disease states: (B_0_) *n*=56, 1845±247 (mean±s.d.); (E) *n*=41, 1357±136; (T) *n*=67, 1643±127; (R) *n*=32, 1845±151; and (B_1_) *n*=41, 1449±212 (Supplementary Table S3).

**Figure 1:**
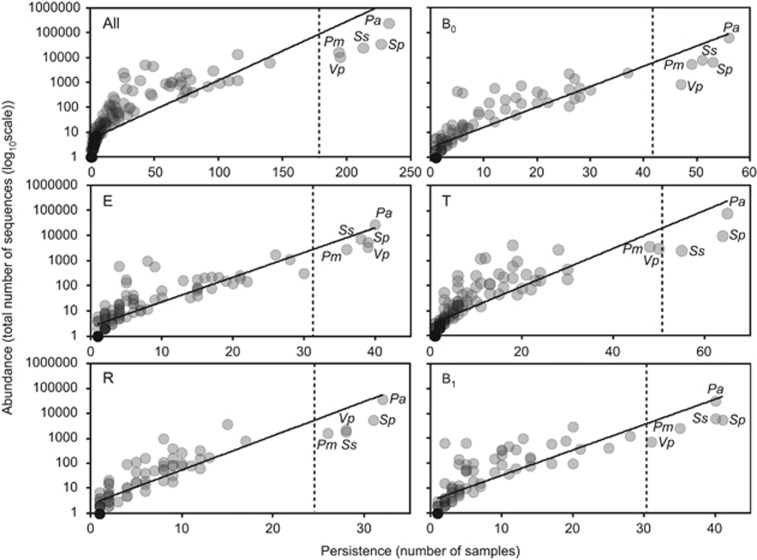
The persistence and abundance (total number of sequence reads) of bacteria taxa present in all longitudinal samples and within each of the five partitioned periods. (All) All samples collected from the 12 CF patients, irrespective of disease state, within the study (*n*=237, *r*^2^=0.7, *F*_1,182_=425.7, *P*<0.001); (B_0_) baseline pre-CFPE (*n*=56, *r*^2^=0.8, *F*_1,106_=429.3, *P*<0.001); (E) CFPE, 30 days prior to treatment (*n*=41, *r*^2^=0.8, *F*_1,121_=415.1, *P*<0.001); (T) CFPE treatment period (*n*=67, *r*^2^=0.7, *F*_1,140_=363.3, *P*<0.001); (R) recovery, 30 days post-CFPE treatment (*n*=32, *r*^2^=0.8, *F*_1,86_=316.7, *P*<0.001); and (B_1_) baseline post-CFPE (*n*=41, *r*^2^=0.7, *F*_1,92_=221.1, *P*<0.001). Core OTUs were defined as those that fell within the upper quartile (dashed lines), and rare OTUs defined as those that did not. Taxa defined as core across all samples are labelled in each panel: *Prevotella melaninogenica* (*Pm*), *Pseudomonas aeruginosa* (*Pa*), *Streptococcus pneumoniae* group (*Sp*), *Streptococcus sanguinis* group (*Ss*), and *Veillonella parvula* (*Vp*).

We have previously established that categorisation of component species in CF microbiota into core and rare species revealed important aspects of species-abundance distributions with a metacommunity that would be neglected without such a distinction (van der Gast *et al.*, 2011, 2014). A coherent metacommunity could be expected to exhibit a direct relationship between prevalence and proportional abundance of individual species within the constituent communities. Consistent with this prediction, the abundance of OTUs, across all samples and within specific disease periods (Figure 1), was significantly correlated with the number of individual respiratory sample communities that those OTUs occupied. On that basis, OTUs present in more than 75% of all samples, and hence highly abundant, were classified as core (Hedin *et al.*, 2015). These were derived from five common taxa, *P. aeruginosa*, *Streptococcus pneumoniae* group, *Streptococcus sanguinis* group, *Prevotella melaninogenica/veroralis/histicola* (abbreviated hereafter as *P. melaninogenica*), and *Veillonella parvula/rogosae* (abbreviated as *P. parvula*), and accounted for over 84% of the total sequence abundance. Conversely, the rare OTU group accounted for the majority of the diversity, 178 OTUs.

**Figure 2:**
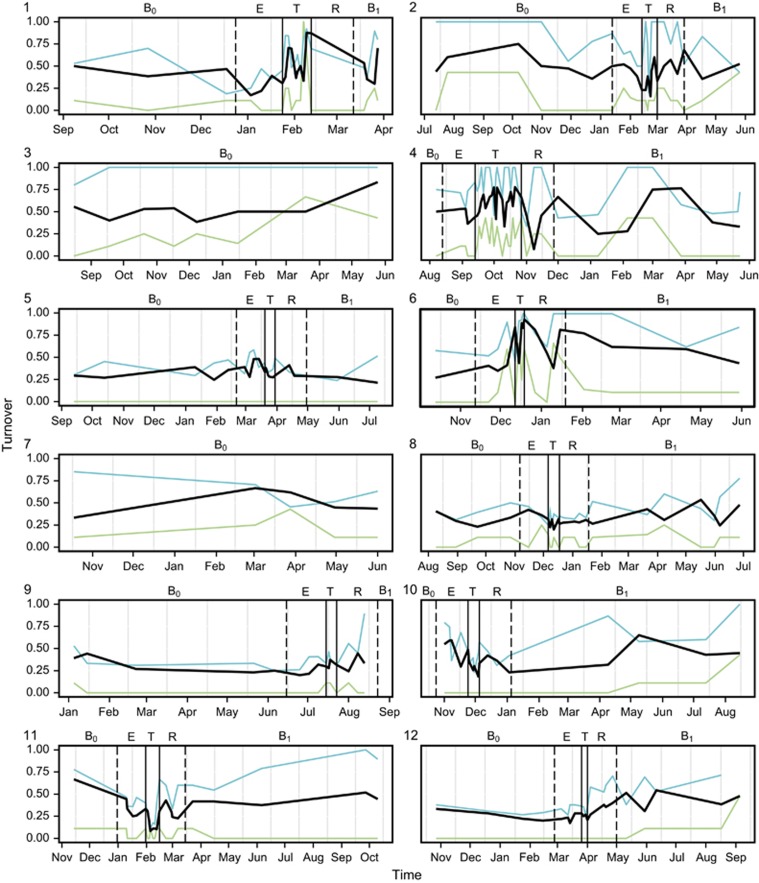
Changes in the rate of OTU turnover for each patient over time. Solid vertical lines indicate the start and end of treatment for CFPE, and dashed lines indicate the start and end of the 30-day period either side of the treatment period. Disease states are denoted by (B_0_) baseline pre-CFPE; (E) CFPE, 30 days prior to treatment; (T) treatment for clinical exacerbation; (R) recovery, 30 days post-CFPE treatment; and (B_1_) stable post-CFPE. Black lines represent turnover in the whole microbiota, while green and blue lines represent turnover within the core and rare OTUs groups, respectively.

**Figure 3:**
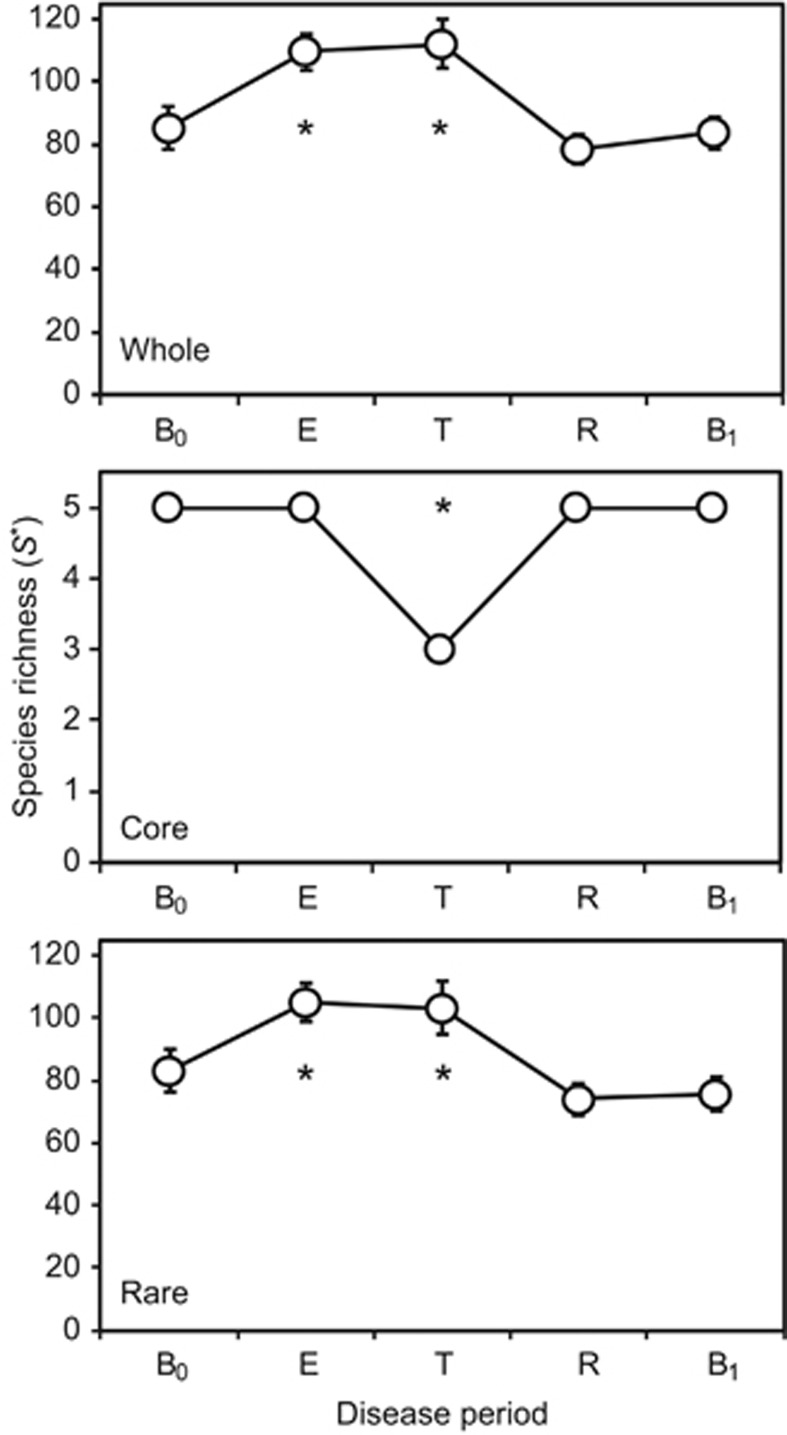
Changes in metacommunity diversity across the disease periods. Given are the whole microbiota, core and rare OTUs groups. Disease periods are denoted by (B_0_) baseline pre-CFPE; (E) CFPE, 30 days prior to treatment; (T) treatment for clinical exacerbation; (R) recovery, 30 days post-CFPE treatment; and (B_1_) baseline post-CFPE. Richness (S*) was calculated with a uniform re-sample size following 1000 iterations in each instance; re-sample sizes by group were *n*=55 656 (whole), *n*=47 092 (core), and *n*=8712 (rare) equating to the lowest number of sequences by disease period within each group. Error bars represent the standard deviation of the mean. Asterisks denote significant differences between B_0_ and subsequent disease periods at the *P*<0.05 level and determined by two sample *t*-tests.

The rate of species turnover, the number of OTUs eliminated and replaced over time, was assessed for each patient over the study period (Figure 2). The rate of OTU turnover showed more variation in the periods surrounding and during treatment for CFPE. Whereas pre- and post-baseline periods had relatively consistent OTU turnover rates, particularly evident for patients 3 and 7 who did not experience a CFPE over the course of the study (Figure 2). The rate of turnover in the rare microbiota group was greater and more variable than that observed for the core microbiota group, suggesting that the more diverse rare microbiota was driving the observed changes in the whole microbiota. Species richness (S*) within the whole microbiota increased significantly from baseline (B_0_) within the CFPE and treatment periods, returning to B_0_ levels in the subsequent recovery and post-CFPE baseline (B_1_) periods. Again, these patterns of diversity were driven by changes within the rare microbiota (Figure 3). Diversity within the core microbiota significantly decreased within the treatment period, with *Prevotella melaninogenica* and *Veillonella parvula* moving into the rare group for that period (Figures 1 and 3).

ANOSIM, using the Bray–Curtis quantitative index of similarity, was used to determine how composition within the whole, core, and rare microbiota changed by disease period (Table 2). No significant change was observed between disease periods within the whole microbiota. Conversely, significant differences within the core microbiota were observed between pre-CFPE baseline and treatment periods (Table 2). Additionally, significant differences within the rare microbiota were observed between pre-CFPE baseline and the treatment and recovery periods. No significant compositional differences were observed between pre- and post-CFPE baseline periods for both the core and rare OTU groups (Table 2). The switching of the two core OTUs, *P*. *melaninogenica* and *Veillonella parvula*, to the rare group in the treatment period will have likely contributed to the significant differences for the core and rare microbiota between pre-CFPE baseline and treatment periods but not for the whole microbiota, where group membership is not accounted for. SIMPER analysis was employed to assess the contribution of individual OTUs to differences in similarity within the whole microbiota, core, and rare OTU groups between disease periods (Supplementary Tables S4–S6). *P. aeruginosa*, followed by *S. pneumoniae* group, contributed most to dissimilarity in the whole microbiota and core OTU group between all disease periods (Supplementary Tables S4 and S5). Within the rare OTU group, *Porphyromonas catoniae* was observed to provide the greatest contribution to dissimilarity between all disease periods, with the exception of the comparison between the CFPE treatment and recovery periods, where *V. parvula* provided the largest contribution.

**Figure 4:**
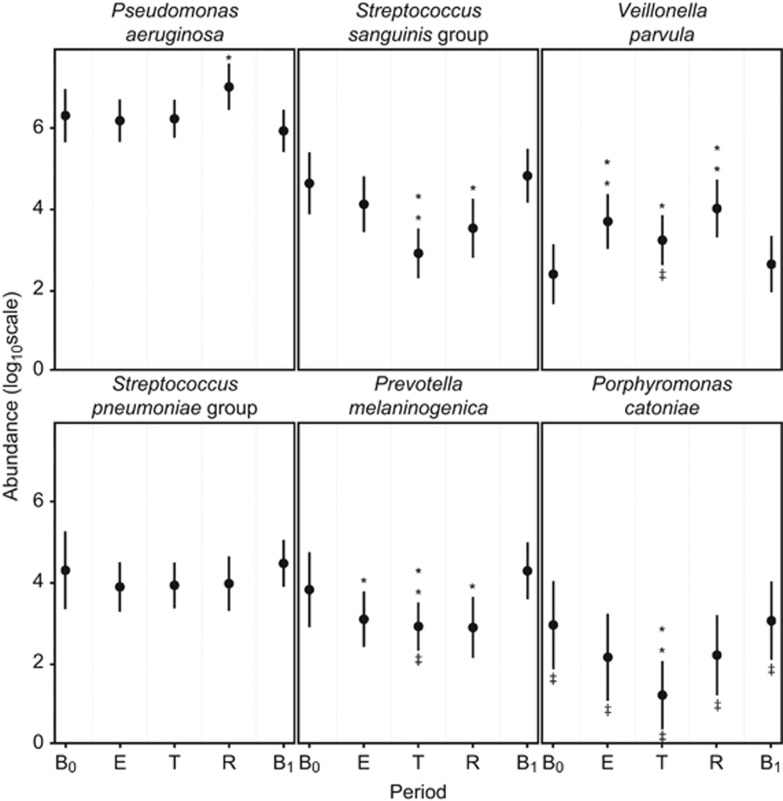
Trends in the relative abundance of bacterial species with change in disease period. Parameters are extracted from mixed-effects models based on 237 samples from 12 patients. (B_0_) baseline pre-CFPE, (E) CFPE, 30 days prior to antibiotic treatment, (T) period of time patients were receiving treatment for pulmonary exacerbation, (R) recovery, 30 days post-treatment, (B_1_) baseline post-CFPE. **P<0.001, *P<0.05. ^‡^ indicates rare group membership.

SIMPER analysis also revealed changes in the percentage contribution of individual OTUs within the core microbiota by disease period. Mixed effects models were used to further investigate how individual core OTUs changed between disease periods (Figure 4). *P. catoniae*, the most abundant of the rare OTU group, was also included in this analysis. These models allowed for the inclusion of both fixed (disease period) and random effects (patient), and therefore variation between patients was accounted for in each model. For *P. aeruginosa*, no significant change in relative abundance from pre-CFPE baseline (B_0_) was observed in the CFPE and treatment periods. However, a significant increase in proportional abundance was observed within the recovery period returning to B_0_ abundance levels in the post-CFPE baseline period (B_1_). No significant changes in relative abundance from B_0_ were observed for the *S. pneumoniae* group OTU in any of the disease periods. *S*. *sanguinis* group and *P*. *catoniae* both had significantly reduced abundance within the treatment period, with the former OTU also significantly lower in the recovery period. *P. melaninogenica* proportional abundance significantly reduced from baseline (B_0_) over the CFPE, treatment, and recovery periods before returning to pre-CFPE baseline (B_0_) levels in period B_1_. Conversely, the proportional abundance of *V. parvula* significantly increased over the same periods (Figure 4).

## Discussion

Cross-sectional studies have informed our understanding of CF chronic lung infections, revealing a complex and highly variable microbiota in a constantly perturbed ecosystem (van der Gast *et al.*, 2011, 2014). However, in order to better understand the progressive nature of chronic lung infection in CF, it is important to investigate how the bacterial communities change through time in relation to disease state and progression. A small number of studies have attempted to understand bacterial community dynamics within the CF lung (Stressmann *et al.*, 2012; Zhao *et al.*, 2012; Carmody *et al.*, 2013; Price *et al.*, 2013). A common limitation, as stated by Carmody and colleagues (2013) for their own study, has been narrow frequency of sampling, which would not allow fluctuations in airway microbiota to be accounted for, within or between clinical periods. Indeed this within clinical period fluctuation was particularly highlighted in the current study, where species turnover was more variable in the CFPE, treatment, and recovery periods (Figure 2). By collecting samples and associated metadata at more frequent intervals the current study was able to not only account for variation within the microbiota over time but also investigate how this related to changes in disease state through the full cycle of an exacerbation event.

Resistance can be defined as ‘the degree to which microbial composition remains unchanged in the face of a disturbance' and resilience as ‘the rate at which microbial composition returns to its original composition after being disturbed' regardless of the system studied (Allison and Martiny, 2008). Previously, Price *et al.* (2013) and Stressmann *et al.* (2011) reported no significant change in community composition through CFPE and treatment. Congruent with those studies we found that the whole microbiota did not significantly change in composition across the five clinical periods studied, indicating resistance to perturbations within the lung (Table 2). However, partitioning the whole chronic lung infection microbiota revealed there was resilience to intervention with antibiotics within both the core and rare OTU groups (Table 2), a pattern also reflected within the diversity based analysis (Figure 3).

Infection with *P. aeruginosa* has been highlighted as a key factor in poor clinical outcomes and is typically one of the main targets for treatment. It was therefore surprising that its proportional abundance remained high across all clinical periods, even exhibiting a significant increase in abundance in the recovery period (Figure 4). While the relative abundance from high-throughput sequencing data of a given species may not be considered strictly quantitative, Price *et al.* (2013) demonstrated a highly significant correlation between the results of *P. aeruginosa*-targeted quantitative polymerase chain reaction and sequencing data. One possible explanation for the increase in *P. aeruginosa* proportional abundances observed after the conclusion of treatment is that antibiotic therapy led to a reduction in the abundance of other species, thereby allowing *P. aeruginosa* to take advantage of newly available physical niche space. Even so, the proportional abundance of *P. aeruginosa* returned to baseline after the 30-day post-treatment recovery period, which may be due to the resilience of the members of the rare species group.

The lack of a generally applicable definition of CFPE and a robust general marker of disease state represents a major challenge for the timely treatment of exacerbations. As stated by Rosenfeld *et al.* (2001) and confirmed from the FEV_1_ data (Supplementary Figure S1), changes in lung function demonstrates little sensitivity or specificity of CFPE diagnosis. Molecular diagnostic tests are being increasingly employed in diagnosis of infectious diseases, allowing disease-associated biomarkers to be used to identify, predict, and monitor changes in infections (Pattison *et al.*, 2013). The identification of biomarkers in CF could potentially provide important tools to improve CFPE identification and therefore improve outcomes for CF patients. Within the current study, *Veillonella* species related to *V. parvula/rogosae* and *Prevotella* species related to *P. melaninogenica/veroralis/histicola* represent potential biomarkers of disease state due to their significant changes in abundance from baseline prior to the start of antibiotic treatment (Figure 4). Development of targeted quantitative polymerase chain reaction based analysis is required to test the efficacy of these two OTUs as molecular diagnostic tools for the onset of CFPE.

While our study was limited to a detailed examination of a relatively small number of patients, our findings have relevance to current management strategies for CFPEs. CF patients receive frequent and prolonged courses of antibiotics, often at high doses, especially for CFPE treatment. Smyth and Elborn (2008) stated there is no comparable medical condition where so many individuals are exposed to antibiotics and risk of cumulative antibiotic side effects in this way. While the resilience within the core and rare species groups will be of concern with regard to treatment, of particular interest to clinicians will be the lack of substantial negative impact on *P. aeruginosa*. Antibiotics administered in response to the worsening symptoms associated with CFPE are expected to target *P. aeruginosa*, which is the emblematic species of chronic lung infections in CF patients. Antimicrobial resistance by recognised respiratory pathogens and other members of the microbiota may be a significant contributing factor underpinning the findings of this study (Sherrard *et al.*, 2014). Therefore this study strongly highlights the merits of continual reassessment of existing antimicrobial treatment regimens and antibiotic delivery strategies (Sherrard *et al.*, 2014).

From a more fundamental perspective, the composition of the bacterial microbiota is most likely not the whole story. At the population level, studies have shown a variety of co-infection interactions between *P. aeruginosa* and other co-colonizing microbes, increasing the virulence of this core CF pathogen. For example, *P. aeruginosa* can use peptidoglycan shed by Gram-positive bacteria as a cue for enhanced virulence (Korgaonkar *et al.*, 2013), metabolites are transferred (e.g. 2,3-butanediol) between fermenting bacteria and *P. aeruginosa* (Venkataraman *et al.*, 2014; Whiteson *et al.*, 2014), and, similarly, ethanol produced by the fungus *Candida albicans* can influence *P. aeruginosa* biofilm formation and phenazine production (Chen *et al.*, 2014). Therefore the wider microbial community and the myriad of interactions therein need to be considered through, for example, metagenomic, metatranscriptomic, and metabolomic approaches to elucidate the roles of bacteria, fungi, and respiratory viruses in tandem, alongside host immune responses. Studies of this nature could reveal the underlying cause or causes of CFPE and it may well be the case that there is no consistent factor (infection or host) that trigger exacerbations, either within or between patients.

## Data deposition

The sequence data reported in this paper have been deposited in the European Nucleotide Archive under study accession numbers ERP005251 and ERP007059, and sample accession numbers ERS421603 and ERS551400.

## Figures and Tables

**Table 1:** Summary of clinical characteristics for individual patients

*Patient*	*Age (years)*	*Gender*	*CFTR genotype*	*BMI*	*CF diabetes*	*CFPE antibiotics*^a^
1	30	Male	ΔF508/NK	29	No	Ciprofloxacin p.o.
2	45	Female	ΔF508/NK	18.2	Yes	Colomycin i.v.+Tobramycin i.v.
3	47	Male	ΔF508/NK	19.9	Yes	
4	22	Female	ΔF508/ΔF508	18	No	Cirprofloxacin p.o., then, Meropenem i.v.+Amakacin i.v.
5	55	Male	ΔF508/G58E	23.9	No	Ceftazidime i.v.+Gentamicin i.v.
6	21	Female	ΔF508/ΔF508	20.3	No	Ciprofloxacin p.o.
7	40	Male	ΔF508/ΔF508	19.4	Yes	
8	22	Male	ΔF508/ΔF508	18.4	Yes	Meropenem i.v.+Colomycin i.v.
9	17	Female	ΔF508/ΔF508	22.5	No	Ceftazidime i.v.+Gentamicin i.v.
10	24	Female	ΔF508/G542X	21	No	Clarithromycin p.o.
11	20	Male	ΔF508/ΔF508	20.4	No	Ciprofloxacin p.o.+Metronidazole
12	20	Male	ΔF508/ΔF508	28.5	No	Ceftazidime i.v.+Gentamicin i.v.

Abbreviations: BMI, body mass index (kg m^2^); CFTR, cystic fibrosis transmembrane conductance regulator; NK, genotype not known (the clinical and functional translation of CFTR (CFTR2); http://cftr2.org).

a Antibiotics administered as intervention for a clinically defined CFPE: p.o., oral; i.v., intravenous.

**Table 2:** Analysis of similarities (ANOSIM) of whole, common, and rare microbiota between disease periods

	*B_0_*	*E*	*T*	*R*	*B_1_*
*Whole*					
B_0_	—	0.210	0.092	0.413	0.562
E	0.012	—	0.276	0.177	0.342
T	0.019	0.012	—	0.778	0.396
R	0.001	0.017	−0.034	—	0.199
B_1_	−0.007	0.001	0.003	0.014	—
					
*Common*					
B_0_	—	0.197	0.01*	0.477	0.533
E	0.011	—	0.04*	0.208	0.488
T	0.145	0.157	—	0.518	0.152
R	−0.005	0.013	−0.008	—	0.140
B_1_	−0.006	−0.004	0.029	0.022	—
					
*Rare*					
B_0_	—	0.123	0.0001*	0.017*	0.574
E	0.027	—	0.0002*	0.024*	0.005*
T	0.195	0.152	—	0.0002*	0.0001*
R	0.194	0.156	0.206	—	0.008*
B_1_	−0.007	0.149	0.194	0.071	—

ANOSIM test statistic (*R*) and probability (*P*) that two compared groups are significantly different at the *P*<0.05 level (denoted with asterisks) are given in the lower and upper triangles, respectively. ANOSIM *R* and *P* values were generated using the Bray–Curtis measure of similarity. Disease states are denoted by (B_0_) stable pre-CFPE; (E) CFPE, 30 days prior to CFPE treatment; (T) treatment for clinical exacerbation; (R) 30 days post-CFPE treatment; and (B_1_) stable post-CFPE.
