# Supplementary material for: Respiratory microbiota resistance and resilience to pulmonary exacerbation and subsequent antimicrobial intervention
Source: ISME J. 2015 Nov 10;10(5):1081–91. doi: 10.1038/ismej.2015.198 (PMC4820042; doi:10.1038/ismej.2015.198)
Supplement: Supplementary Figure S1 [file ismej2015198x1.pdf]

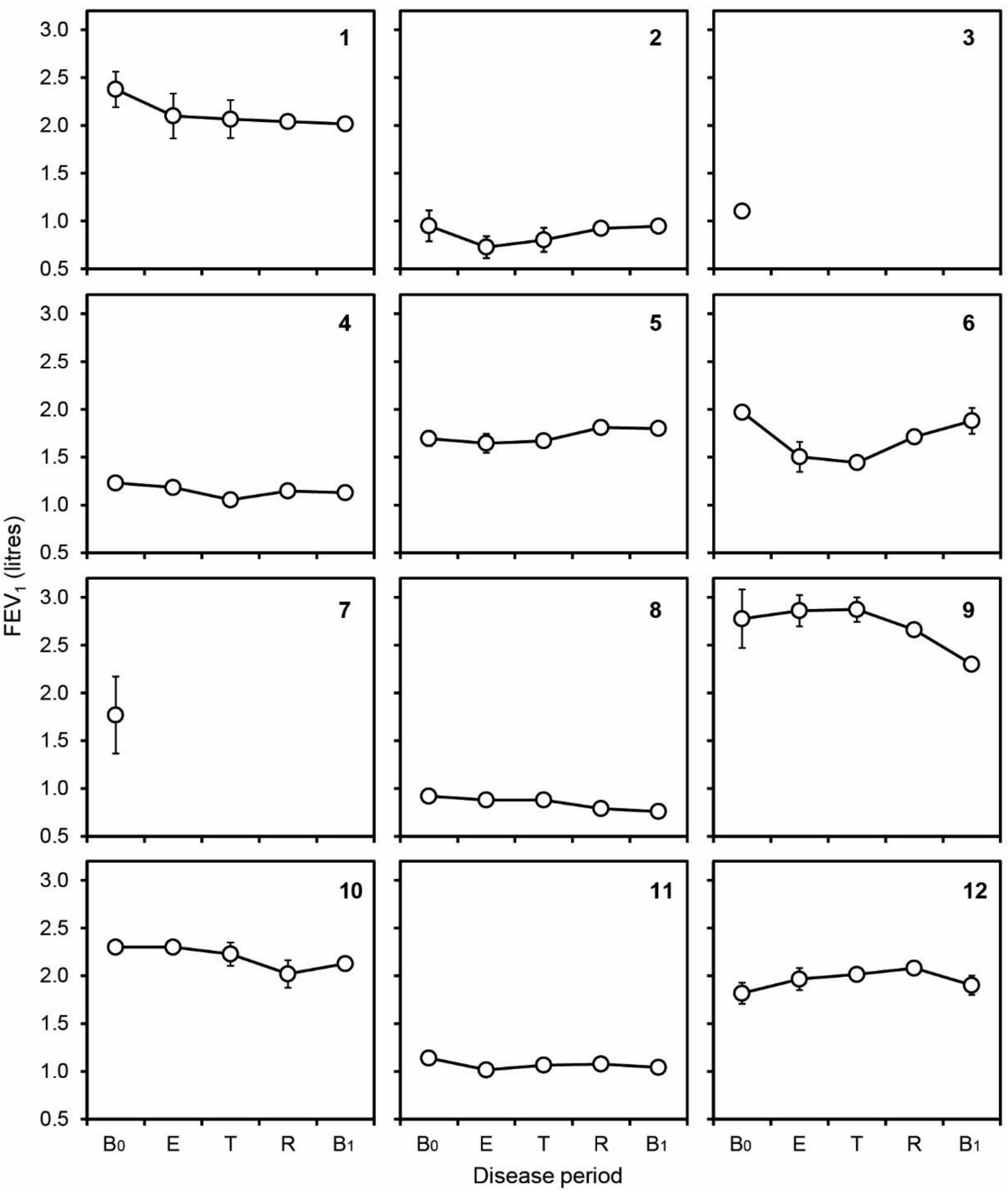

Figure S1 Changes in lung function (FEV_1 in litres) through disease periods for each patient. (B_0) Stable pre-cystic fibrosis pulmonary exacerbation (CFPE), (E) CFPE, 30 days prior to treatment, (T) CFPE treatment, (R) recovery, 30 days post-CFPE treatment, and (B_1) stable post-CFPE. Patients 3 and 7 did not experience CFPE within the study, therefore only lung function data for disease period B_0 are given. In each instance, mean FEV_1 values are given per disease period. Error bars represent the standard deviation of the mean.
