# Supplementary material for: Respiratory microbiota resistance and resilience to pulmonary exacerbation and subsequent antimicrobial intervention
Source: ISME J. 2015 Nov 10;10(5):1081–91. doi: 10.1038/ismej.2015.198 (PMC4820042; doi:10.1038/ismej.2015.198)
Supplement: Supplementary Table S1 [file ismej2015198x2.doc]

**Table S1** A list of barcodes used with their associated samples are given below. The raw sequence data reported in this paper have been deposited in the European Nucleotide Archive under study accession numbers ERP005251 and ERP007059, and sample accession numbers ERS421603 and ERS551400.

| **Sanger Barcode ID** | **Barcode** | **Sputum ID** | **Sample No** | **Patient** | **Clinical Period** | **Study accession number** | **Sample accession number** |
| --- | --- | --- | --- | --- | --- | --- | --- |
| GolayBarcode149 | CAACTCATCGTA | 01_006 | 6 | 1 | B0 | ERP007059 | ERS551400 |
| GolayBarcode461 | TACTACATGGTC | 01_031 | 31 | 1 | B0 | ERP007059 | ERS551400 |
| GolayBarcode129 | ATGACCATCGTG | 01_055 | 55 | 1 | B0 | ERP007059 | ERS551400 |
| GolayBarcode67 | AGAGTCCTGAGC | 01_075 | 75 | 1 | B0 | ERP007059 | ERS551400 |
| GolayBarcode48 | ACTATTGTCACG | 01_078 | 78 | 1 | E | ERP005251 | ERS421603 |
| GolayBarcode60 | AGAACACGTCTC | 01_081 | 81 | 1 | E | ERP005251 | ERS421603 |
| GolayBarcode72 | AGCACACCTACA | 01_084 | 84 | 1 | E | ERP007059 | ERS551400 |
| GolayBarcode84 | AGCTCTCAGAGG | 01_087 | 87 | 1 | E | ERP005251 | ERS421603 |
| GolayBarcode96 | AGTCTCGCATAT | 01_089 | 89 | 1 | T | ERP005251 | ERS421603 |
| GolayBarcode120 | ATCGATCTGTGG | 01_091 | 91 | 1 | T | ERP005251 | ERS421603 |
| GolayBarcode112 | ATAGGCGATCTC | 01_093 | 93 | 1 | T | ERP005251 | ERS421603 |
| GolayBarcode104 | AGTTCTACGTCA | 01_095 | 95 | 1 | T | ERP005251 | ERS421603 |
| GolayBarcode479 | TAGTGCTGCGTA | 01_096 | 96 | 1 | T | ERP005251 | ERS421603 |
| GolayBarcode150 | CAAGATCGACTC | 01_097 | 97 | 1 | T | ERP005251 | ERS421603 |
| GolayBarcode143 | ATGTGCACGACT | 01_099 | 99 | 1 | T | ERP005251 | ERS421603 |
| GolayBarcode135 | ATGCCTGAGCAG | 01_100 | 100 | 1 | T | ERP005251 | ERS421603 |
| GolayBarcode421 | GTCGCTGTCTTC | 01_101 | 101 | 1 | T | ERP007059 | ERS551400 |
| GolayBarcode103 | AGTTCAGACGCT | 01_112 | 112 | 1 | R | ERP005251 | ERS421603 |
| GolayBarcode119 | ATCCTCAGTAGT | 01_114 | 114 | 1 | B1 | ERP007059 | ERS551400 |
| GolayBarcode111 | ATAGCTCCATAC | 01_116 | 116 | 1 | B1 | ERP005251 | ERS421603 |
| GolayBarcode478 | TAGTCGTCTAGT | 01_117 | 117 | 1 | B1 | ERP005251 | ERS421603 |
| GolayBarcode20 | ACATCACTTAGC | 02_003 | 3 | 2 | B0 | ERP007059 | ERS551400 |
| GolayBarcode39 | ACGGTGAGTGTC | 02_020 | 20 | 2 | B0 | ERP007059 | ERS551400 |
| GolayBarcode88 | AGGCTACACGAC | 02_022 | 22 | 2 | B0 | ERP007059 | ERS551400 |
| GolayBarcode51 | ACTCGATTCGAT | 02_051 | 51 | 2 | B0 | ERP007059 | ERS551400 |
| GolayBarcode470 | TAGCACACCTAT | 02_061 | 61 | 2 | B0 | ERP007059 | ERS551400 |
| GolayBarcode185 | CATATACTCGCA | 02_073 | 73 | 2 | B0 | ERP007059 | ERS551400 |
| GolayBarcode104 | AGTTCTACGTCA | 02_084 | 84 | 2 | B0 | ERP007059 | ERS551400 |
| GolayBarcode59 | ACTTGTAGCAGC | 02_090 | 90 | 2 | B0 | ERP007059 | ERS551400 |
| GolayBarcode97 | AGTGAGAGAAGC | 02_095 | 95 | 2 | E | ERP005251 | ERS421603 |
| GolayBarcode105 | ATAATCTCGTCG | 02_098 | 98 | 2 | E | ERP005251 | ERS421603 |
| GolayBarcode113 | ATATCGCTACTG | 02_101 | 101 | 2 | E | ERP005251 | ERS421603 |
| GolayBarcode121 | ATCGCGGACGAT | 02_103 | 103 | 2 | E | ERP005251 | ERS421603 |
| GolayBarcode128 | ATCTTAGACTGC | 02_106a | 106 | 2 | T | ERP005251 | ERS421603 |
| GolayBarcode430 | GTCTTCGTCGCT | 02_108 | 108 | 2 | T | ERP007059 | ERS551400 |
| GolayBarcode136 | ATGCGTAGTGCG | 02_109a | 109 | 2 | T | ERP005251 | ERS421603 |
| GolayBarcode144 | ATGTGTCGACTT | 02_111 | 111 | 2 | T | ERP005251 | ERS421603 |
| GolayBarcode473 | TAGCGACATCTG | 02_112 | 112 | 2 | T | ERP005251 | ERS421603 |
| GolayBarcode98 | AGTGCGATGCGT | 02_115 | 115 | 2 | T | ERP005251 | ERS421603 |
| GolayBarcode106 | ATACACGTGGCG | 02_118 | 118 | 2 | T | ERP005251 | ERS421603 |
| GolayBarcode114 | ATATGCCAGTGC | 02_122 | 122 | 2 | R | ERP005251 | ERS421603 |
| GolayBarcode122 | ATCGCTCGAGGA | 02_125 | 125 | 2 | R | ERP005251 | ERS421603 |
| GolayBarcode130 | ATGACTCATTCG | 02_128 | 128 | 2 | R | ERP005251 | ERS421603 |
| GolayBarcode71 | AGATGTTCTGCT | 02_131 | 131 | 2 | B1 | ERP007059 | ERS551400 |
| GolayBarcode47 | ACTAGCTCCATA | 02_137 | 137 | 2 | B1 | ERP007059 | ERS551400 |

Table S1 continued.

| **Sanger Barcode ID** | **Barcode** | **Sputum ID** | **Sample No** | **Patient** | **Clinical Period** | **Study accession number** | **Sample accession number** |
| --- | --- | --- | --- | --- | --- | --- | --- |
| GolayBarcode188 | CATCATGAGGCT | 02_153 | 153 | 2 | B1 | ERP007059 | ERS551400 |
| GolayBarcode455 | TACCGCTAGTAG | 03_017 | 17 | 3 | B0 | ERP007059 | ERS551400 |
| GolayBarcode146 | ATTCTGTGAGCG | 03_030 | 30 | 3 | B0 | ERP007059 | ERS551400 |
| GolayBarcode466 | TAGACTGTACTC | 03_040 | 40 | 3 | B0 | ERP007059 | ERS551400 |
| GolayBarcode124 | ATCTACTACACG | 03_054 | 54 | 3 | B0 | ERP007059 | ERS551400 |
| GolayBarcode142 | ATGTCACCGTGA | 03_063 | 63 | 3 | B0 | ERP007059 | ERS551400 |
| GolayBarcode474 | TAGCGGATCACG | 03_072 | 72 | 3 | B0 | ERP007059 | ERS551400 |
| GolayBarcode427 | GTCTCTCTACGC | 03_085 | 85 | 3 | B0 | ERP007059 | ERS551400 |
| GolayBarcode66 | AGAGTAGCTAAG | 03_110 | 110 | 3 | B0 | ERP007059 | ERS551400 |
| GolayBarcode444 | GTTCGCGTATAG | 03_135 | 135 | 3 | B0 | ERP007059 | ERS551400 |
| GolayBarcode445 | GTTGACGACAGC | 05_002 | 2 | 4 | B0 | ERP007059 | ERS551400 |
| GolayBarcode458 | TACGGTATGTCT | 05_012 | 12 | 4 | B0 | ERP007059 | ERS551400 |
| GolayBarcode82 | AGCTATCCACGA | 05_014 | 14 | 4 | E | ERP005251 | ERS421603 |
| GolayBarcode32 | ACGATGCGACCA | 05_016 | 16 | 4 | E | ERP007059 | ERS551400 |
| GolayBarcode70 | AGATCTCTGCAT | 05_018 | 18 | 4 | E | ERP005251 | ERS421603 |
| GolayBarcode58 | ACTGTGACTTCA | 05_021 | 21 | 4 | T | ERP005251 | ERS421603 |
| GolayBarcode46 | ACTACGTGTGGT | 05_023 | 23 | 4 | T | ERP005251 | ERS421603 |
| GolayBarcode34 | ACGCGATACTGG | 05_024 | 24 | 4 | T | ERP005251 | ERS421603 |
| GolayBarcode22 | ACATGTCACGTG | 05_026 | 26 | 4 | T | ERP005251 | ERS421603 |
| GolayBarcode10 | ACACGAGCCACA | 05_028 | 28 | 4 | T | ERP005251 | ERS421603 |
| GolayBarcode93 | AGTCACATCACT | 05_030 | 30 | 4 | T | ERP005251 | ERS421603 |
| GolayBarcode81 | AGCGTAGGTCGT | 05_034 | 34 | 4 | T | ERP005251 | ERS421603 |
| GolayBarcode69 | AGATCGGCTCGA | 05_036 | 36 | 4 | T | ERP005251 | ERS421603 |
| GolayBarcode57 | ACTGTCGAAGCT | 05_038 | 38 | 4 | T | ERP005251 | ERS421603 |
| GolayBarcode45 | ACTACAGCCTAT | 05_040 | 40 | 4 | T | ERP005251 | ERS421603 |
| GolayBarcode33 | ACGCAACTGCTA | 05_041 | 41 | 4 | T | ERP005251 | ERS421603 |
| GolayBarcode450 | TACACACATGGC | 05_044 | 44 | 4 | T | ERP007059 | ERS551400 |
| GolayBarcode9 | ACACATGTCTAC | 05_045 | 45 | 4 | T | ERP005251 | ERS421603 |
| GolayBarcode92 | AGTAGTATCCTC | 05_047 | 47 | 4 | T | ERP005251 | ERS421603 |
| GolayBarcode80 | AGCGCTGATGTG | 05_049 | 49 | 4 | T | ERP005251 | ERS421603 |
| GolayBarcode68 | AGATACACGCGC | 05_051 | 51 | 4 | T | ERP005251 | ERS421603 |
| GolayBarcode56 | ACTGTACGCGTA | 05_053 | 53 | 4 | T | ERP005251 | ERS421603 |
| GolayBarcode44 | ACGTTAGCACAC | 05_055 | 55 | 4 | T | ERP005251 | ERS421603 |
| GolayBarcode32 | ACGATGCGACCA | 05_060 | 60 | 4 | R | ERP005251 | ERS421603 |
| GolayBarcode20 | ACATCACTTAGC | 05_062 | 62 | 4 | R | ERP005251 | ERS421603 |
| GolayBarcode8 | ACACACTATGGC | 05_065 | 65 | 4 | R | ERP007059 | ERS551400 |
| GolayBarcode91 | AGTACTGCAGGC | 05_068 | 68 | 4 | R | ERP005251 | ERS421603 |
| GolayBarcode7 | AATCGTGACTCG | 05_079 | 79 | 4 | B1 | ERP007059 | ERS551400 |
| GolayBarcode108 | ATACGTCTTCGA | 05_088 | 88 | 4 | B1 | ERP007059 | ERS551400 |
| GolayBarcode60 | AGAACACGTCTC | 05_098 | 98 | 4 | B1 | ERP007059 | ERS551400 |
| GolayBarcode443 | GTTAGAGCACTC | 05_112 | 112 | 4 | B1 | ERP007059 | ERS551400 |
| GolayBarcode44 | ACGTTAGCACAC | 05_125 | 125 | 4 | B1 | ERP007059 | ERS551400 |
| GolayBarcode72 | AGCACACCTACA | 05_130 | 130 | 4 | B1 | ERP007059 | ERS551400 |
| GolayBarcode3 | AACTGTGCGTAC | 05_138 | 138 | 4 | B1 | ERP007059 | ERS551400 |
| GolayBarcode135 | ATGCCTGAGCAG | 05_139 | 139 | 4 | B1 | ERP007059 | ERS551400 |
| GolayBarcode454 | TACATCACCACA | 06_006 | 6 | 5 | B0 | ERP007059 | ERS551400 |
| GolayBarcode36 | ACGCTATCTGGA | 06_021 | 21 | 5 | B0 | ERP007059 | ERS551400 |

Table S1 continued.

| **Sanger Barcode ID** | **Barcode** | **Sputum ID** | **Sample No** | **Patient** | **Clinical Period** | **Study accession number** | **Sample accession number** |
| --- | --- | --- | --- | --- | --- | --- | --- |
| GolayBarcode15 | ACAGAGTCGGCT | 06_030 | 30 | 5 | B0 | ERP007059 | ERS551400 |
| GolayBarcode123 | ATCGTACAACTC | 06_061 | 61 | 5 | B0 | ERP007059 | ERS551400 |
| GolayBarcode76 | AGCATATGAGAG | 06_067 | 67 | 5 | B0 | ERP007059 | ERS551400 |
| GolayBarcode95 | AGTCTACTCTGA | 06_072 | 72 | 5 | B0 | ERP005251 | ERS421603 |
| GolayBarcode94 | AGTCCATAGCTG | 06_079 | 79 | 5 | E | ERP005251 | ERS421603 |
| GolayBarcode11 | ACACGGTGTCTA | 06_081 | 81 | 5 | E | ERP005251 | ERS421603 |
| GolayBarcode116 | ATCACTAGTCAC | 06_082 | 82 | 5 | E | ERP007059 | ERS551400 |
| GolayBarcode23 | ACATTCAGCGCA | 06_083 | 83 | 5 | E | ERP005251 | ERS421603 |
| GolayBarcode35 | ACGCGCAGATAC | 06_086a | 86 | 5 | E | ERP005251 | ERS421603 |
| GolayBarcode59 | ACTTGTAGCAGC | 06_087 | 87 | 5 | T | ERP005251 | ERS421603 |
| GolayBarcode71 | AGATGTTCTGCT | 06_089 | 89 | 5 | T | ERP005251 | ERS421603 |
| GolayBarcode83 | AGCTCCATACAG | 06_090 | 90 | 5 | T | ERP005251 | ERS421603 |
| GolayBarcode12 | ACACTAGATCCG | 06_094 | 94 | 5 | T | ERP005251 | ERS421603 |
| GolayBarcode24 | ACCACATACATC | 06_096 | 96 | 5 | T | ERP005251 | ERS421603 |
| GolayBarcode24 | ACCACATACATC | 06_100 | 100 | 5 | R | ERP007059 | ERS551400 |
| GolayBarcode36 | ACGCTATCTGGA | 06_102 | 102 | 5 | R | ERP005251 | ERS421603 |
| GolayBarcode48 | ACTATTGTCACG | 06_112 | 112 | 5 | B1 | ERP007059 | ERS551400 |
| GolayBarcode31 | ACGAGTGCTATC | 06_125 | 125 | 5 | B1 | ERP007059 | ERS551400 |
| GolayBarcode449 | TAAGCGCAGCAC | 07_015 | 15 | 6 | B0 | ERP007059 | ERS551400 |
| GolayBarcode473 | TAGCGACATCTG | 07_036 | 36 | 6 | B0 | ERP007059 | ERS551400 |
| GolayBarcode78 | AGCGACTGTGCA | 07_053 | 53 | 6 | E | ERP005251 | ERS421603 |
| GolayBarcode66 | AGAGTAGCTAAG | 07_056 | 56 | 6 | E | ERP005251 | ERS421603 |
| GolayBarcode54 | ACTGACAGCCAT | 07_059 | 59 | 6 | E | ERP005251 | ERS421603 |
| GolayBarcode42 | ACGTGAGAGAAT | 07_062 | 62 | 6 | E | ERP005251 | ERS421603 |
| GolayBarcode18 | ACAGTGCTTCAT | 07_065 | 65 | 6 | T | ERP005251 | ERS421603 |
| GolayBarcode193 | CATGGCTACACA | 07_066 | 66 | 6 | T | ERP007059 | ERS551400 |
| GolayBarcode6 | AATCAGTCTCGT | 07_067 | 67 | 6 | T | ERP005251 | ERS421603 |
| GolayBarcode89 | AGGTGTGATCGC | 07_068 | 68 | 6 | T | ERP005251 | ERS421603 |
| GolayBarcode77 | AGCCATACTGAC | 07_071 | 71 | 6 | R | ERP005251 | ERS421603 |
| GolayBarcode53 | ACTCTTCTAGAG | 07_073 | 73 | 6 | R | ERP005251 | ERS421603 |
| GolayBarcode65 | AGAGCAAGAGCA | 07_075 | 75 | 6 | R | ERP005251 | ERS421603 |
| GolayBarcode460 | TACTAATCTGCG | 07_077 | 77 | 6 | R | ERP007059 | ERS551400 |
| GolayBarcode126 | ATCTGAGCTGGT | 07_085 | 85 | 6 | B1 | ERP007059 | ERS551400 |
| GolayBarcode191 | CATGAGTGCTAC | 07_094 | 94 | 6 | B1 | ERP007059 | ERS551400 |
| GolayBarcode136 | ATGCGTAGTGCG | 07_122 | 122 | 6 | B1 | ERP007059 | ERS551400 |
| GolayBarcode448 | TAACTCTGATGC | 07_137 | 137 | 6 | B1 | ERP007059 | ERS551400 |
| GolayBarcode478 | TAGTCGTCTAGT | 08_022 | 22 | 7 | B0 | ERP007059 | ERS551400 |
| GolayBarcode447 | TAACAGTCGCTG | 08_033 | 33 | 7 | B0 | ERP007059 | ERS551400 |
| GolayBarcode467 | TAGAGAGAGTGG | 08_086 | 86 | 7 | B0 | ERP007059 | ERS551400 |
| GolayBarcode468 | TAGATAGCAGGA | 08_097 | 97 | 7 | B0 | ERP007059 | ERS551400 |
| GolayBarcode464 | TACTTACTGCAG | 08_109 | 109 | 7 | B0 | ERP007059 | ERS551400 |
| GolayBarcode439 | GTGTCTACATTG | 08_121 | 121 | 7 | B0 | ERP007059 | ERS551400 |
| GolayBarcode19 | ACAGTTGCGCGA | 09_005 | 5 | 8 | B0 | ERP007059 | ERS551400 |
| GolayBarcode55 | ACTGATCCTAGT | 09_009 | 9 | 8 | B0 | ERP007059 | ERS551400 |
| GolayBarcode12 | ACACTAGATCCG | 09_017 | 17 | 8 | B0 | ERP007059 | ERS551400 |
| GolayBarcode91 | AGTACTGCAGGC | 09_027 | 27 | 8 | B0 | ERP007059 | ERS551400 |
| GolayBarcode4 | AAGAGATGTCGA | 09_047 | 47 | 8 | B0 | ERP007059 | ERS551400 |

Table S1 continued.

| **Sanger Barcode ID** | **Barcode** | **Sputum ID** | **Sample No** | **Patient** | **Clinical Period** | **Study accession number** | **Sample accession number** |
| --- | --- | --- | --- | --- | --- | --- | --- |
| GolayBarcode138 | ATGGCAGCTCTA | 09_055 | 55 | 8 | E | ERP005251 | ERS421603 |
| GolayBarcode145 | ATTATCGTGCAC | 09_061 | 61 | 8 | E | ERP005251 | ERS421603 |
| GolayBarcode474 | TAGCGGATCACG | 09_064 | 64 | 8 | E | ERP005251 | ERS421603 |
| GolayBarcode99 | AGTGGATGCTCT | 09_066 | 66 | 8 | T | ERP005251 | ERS421603 |
| GolayBarcode107 | ATACAGAGCTCC | 09_068 | 68 | 8 | T | ERP005251 | ERS421603 |
| GolayBarcode115 | ATCACGTAGCGG | 09_071 | 71 | 8 | T | ERP005251 | ERS421603 |
| GolayBarcode123 | ATCGTACAACTC | 09_075 | 75 | 8 | T | ERP005251 | ERS421603 |
| GolayBarcode131 | ATGAGACTCCAC | 09_076 | 76 | 8 | R | ERP005251 | ERS421603 |
| GolayBarcode139 | ATGGCGTGCACA | 09_078 | 78 | 8 | R | ERP005251 | ERS421603 |
| GolayBarcode146 | ATTCTGTGAGCG | 09_080 | 80 | 8 | R | ERP005251 | ERS421603 |
| GolayBarcode475 | TAGCTCGTAACT | 09_082 | 82 | 8 | R | ERP005251 | ERS421603 |
| GolayBarcode100 | AGTGTCACGGTG | 09_085 | 85 | 8 | R | ERP005251 | ERS421603 |
| GolayBarcode92 | AGTAGTATCCTC | 09_087 | 87 | 8 | B1 | ERP007059 | ERS551400 |
| GolayBarcode426 | GTCTCATGTAGG | 09_120 | 120 | 8 | B1 | ERP007059 | ERS551400 |
| GolayBarcode480 | TAGTGTGCTTCA | 09_133 | 133 | 8 | B1 | ERP007059 | ERS551400 |
| GolayBarcode28 | ACCTCGATCAGA | 09_156 | 156 | 8 | B1 | ERP007059 | ERS551400 |
| GolayBarcode96 | AGTCTCGCATAT | 09_160 | 160 | 8 | B1 | ERP007059 | ERS551400 |
| GolayBarcode40 | ACGTACTCAGTG | 09_163 | 163 | 8 | B1 | ERP007059 | ERS551400 |
| GolayBarcode75 | AGCAGTCGCGAT | 09_303 | 303 | 8 | B1 | ERP007059 | ERS551400 |
| GolayBarcode144 | ATGTGTCGACTT | 10_001 | 1 | 9 | B0 | ERP007059 | ERS551400 |
| GolayBarcode186 | CATATCGCAGTT | 10_063 | 63 | 9 | B0 | ERP007059 | ERS551400 |
| GolayBarcode16 | ACAGCAGTGGTC | 10_067 | 67 | 9 | B0 | ERP007059 | ERS551400 |
| GolayBarcode189 | CATCGTATCAAC | 10_083 | 83 | 9 | B0 | ERP007059 | ERS551400 |
| GolayBarcode479 | TAGTGCTGCGTA | 10_124 | 124 | 9 | B0 | ERP007059 | ERS551400 |
| GolayBarcode446 | GTTGTATACTCG | 10_130 | 130 | 9 | B0 | ERP007059 | ERS551400 |
| GolayBarcode28 | ACCTCGATCAGA | 10_138 | 138 | 9 | E | ERP005251 | ERS421603 |
| GolayBarcode40 | ACGTACTCAGTG | 10_141 | 141 | 9 | E | ERP005251 | ERS421603 |
| GolayBarcode52 | ACTCGCACAGGA | 10_144 | 144 | 9 | E | ERP005251 | ERS421603 |
| GolayBarcode64 | AGAGAGCAAGTG | 10_147 | 147 | 9 | T | ERP005251 | ERS421603 |
| GolayBarcode76 | AGCATATGAGAG | 10_149 | 149 | 9 | T | ERP005251 | ERS421603 |
| GolayBarcode88 | AGGCTACACGAC | 10_150 | 150 | 9 | T | ERP005251 | ERS421603 |
| GolayBarcode5 | AAGCTGCAGTCG | 10_153 | 153 | 9 | T | ERP005251 | ERS421603 |
| GolayBarcode17 | ACAGCTAGCTTG | 10_157 | 157 | 9 | R | ERP005251 | ERS421603 |
| GolayBarcode29 | ACCTGTCTCTCT | 10_160 | 160 | 9 | R | ERP005251 | ERS421603 |
| GolayBarcode41 | ACGTCTGTAGCA | 10_162 | 162 | 9 | R | ERP005251 | ERS421603 |
| GolayBarcode131 | ATGAGACTCCAC | 11_006 | 6 | 10 | B0 | ERP007059 | ERS551400 |
| GolayBarcode149 | CAACTCATCGTA | 11_025 | 25 | 10 | E | ERP007059 | ERS551400 |
| GolayBarcode107 | ATACAGAGCTCC | 11_027 | 27 | 10 | E | ERP005251 | ERS421603 |
| GolayBarcode142 | ATGTCACCGTGA | 11_028 | 28 | 10 | E | ERP005251 | ERS421603 |
| GolayBarcode134 | ATGCAGCTCAGT | 11_032 | 32 | 10 | E | ERP005251 | ERS421603 |
| GolayBarcode126 | ATCTGAGCTGGT | 11_035 | 35 | 10 | E | ERP005251 | ERS421603 |
| GolayBarcode118 | ATCCGATCACAG | 11_036 | 36 | 10 | T | ERP005251 | ERS421603 |
| GolayBarcode110 | ATACTCACTCAG | 11_040 | 40 | 10 | T | ERP005251 | ERS421603 |
| GolayBarcode102 | AGTTAGTGCGTC | 11_041 | 41 | 10 | T | ERP005251 | ERS421603 |
| GolayBarcode477 | TAGGTATCTCAC | 11_045 | 45 | 10 | T | ERP005251 | ERS421603 |
| GolayBarcode148 | CAACTATCAGCT | 11_046 | 46 | 10 | T | ERP005251 | ERS421603 |
| GolayBarcode141 | ATGTACGGCGAC | 11_051 | 51 | 10 | R | ERP005251 | ERS421603 |

Table S1 continued

| **Sanger Barcode ID** | **Barcode** | **Sputum ID** | **Sample No** | **Patient** | **Clinical Period** | **Study accession number** | **Sample accession number** |
| --- | --- | --- | --- | --- | --- | --- | --- |
| GolayBarcode133 | ATGCACTGGCGA | 11_055 | 55 | 10 | R | ERP005251 | ERS421603 |
| GolayBarcode125 | ATCTCTGGCATA | 11_057 | 57 | 10 | R | ERP005251 | ERS421603 |
| GolayBarcode453 | TACAGTCTCATG | 11_113 | 113 | 10 | B1 | ERP007059 | ERS551400 |
| GolayBarcode434 | GTGATAGTGCCG | 11_127 | 139 | 10 | B1 | ERP007059 | ERS551400 |
| GolayBarcode132 | ATGATCGAGAGA | 11_154 | 154 | 10 | B1 | ERP007059 | ERS551400 |
| GolayBarcode425 | GTCTATCGGAGT | 11_163 | 163 | 10 | B1 | ERP007059 | ERS551400 |
| GolayBarcode127 | ATCTGGTGCTAT | 12_002 | 2 | 11 | B0 | ERP007059 | ERS551400 |
| GolayBarcode99 | AGTGGATGCTCT | 12_023 | 23 | 11 | B0 | ERP007059 | ERS551400 |
| GolayBarcode50 | ACTCAGATACTC | 12_037 | 37 | 11 | E | ERP005251 | ERS421603 |
| GolayBarcode79 | AGCGAGCTATCT | 12_038 | 38 | 11 | E | ERP007059 | ERS551400 |
| GolayBarcode62 | AGACGTGCACTG | 12_040 | 40 | 11 | E | ERP005251 | ERS421603 |
| GolayBarcode74 | AGCAGCACTTGT | 12_042 | 42 | 11 | E | ERP005251 | ERS421603 |
| GolayBarcode86 | AGCTTGACAGCT | 12_046 | 46 | 11 | E | ERP005251 | ERS421603 |
| GolayBarcode3 | AACTGTGCGTAC | 12_048 | 48 | 11 | T | ERP005251 | ERS421603 |
| GolayBarcode15 | ACAGAGTCGGCT | 12_050 | 50 | 11 | T | ERP005251 | ERS421603 |
| GolayBarcode27 | ACCGCAGAGTCA | 12_051 | 51 | 11 | T | ERP005251 | ERS421603 |
| GolayBarcode39 | ACGGTGAGTGTC | 12_054 | 54 | 11 | T | ERP005251 | ERS421603 |
| GolayBarcode51 | ACTCGATTCGAT | 12_056 | 56 | 11 | T | ERP005251 | ERS421603 |
| GolayBarcode63 | AGACTGCGTACT | 12_058 | 58 | 11 | T | ERP005251 | ERS421603 |
| GolayBarcode75 | AGCAGTCGCGAT | 12_061 | 61 | 11 | T | ERP005251 | ERS421603 |
| GolayBarcode87 | AGGACGCACTGT | 12_064 | 64 | 11 | R | ERP005251 | ERS421603 |
| GolayBarcode4 | AAGAGATGTCGA | 12_066 | 66 | 11 | R | ERP005251 | ERS421603 |
| GolayBarcode56 | ACTGTACGCGTA | 12_067 | 67 | 11 | R | ERP007059 | ERS551400 |
| GolayBarcode16 | ACAGCAGTGGTC | 12_069 | 69 | 11 | R | ERP005251 | ERS421603 |
| GolayBarcode115 | ATCACGTAGCGG | 12_082 | 82 | 11 | B1 | ERP007059 | ERS551400 |
| GolayBarcode8 | ACACACTATGGC | 12_083 | 83 | 11 | B1 | ERP007059 | ERS551400 |
| GolayBarcode52 | ACTCGCACAGGA | 12_108 | 108 | 11 | B1 | ERP007059 | ERS551400 |
| GolayBarcode27 | ACCGCAGAGTCA | 12_110 | 110 | 11 | B1 | ERP007059 | ERS551400 |
| GolayBarcode100 | AGTGTCACGGTG | 12_115 | 115 | 11 | B1 | ERP007059 | ERS551400 |
| GolayBarcode35 | ACGCGCAGATAC | 13_002 | 2 | 12 | B0 | ERP007059 | ERS551400 |
| GolayBarcode103 | AGTTCAGACGCT | 13_011 | 11 | 12 | B0 | ERP007059 | ERS551400 |
| GolayBarcode459 | TACGTGTACGTG | 13_038 | 38 | 12 | B0 | ERP007059 | ERS551400 |
| GolayBarcode63 | AGACTGCGTACT | 13_051 | 51 | 12 | B0 | ERP007059 | ERS551400 |
| GolayBarcode437 | GTGGCGATACAC | 13_060 | 60 | 12 | B0 | ERP007059 | ERS551400 |
| GolayBarcode108 | ATACGTCTTCGA | 13_068 | 68 | 12 | E | ERP005251 | ERS421603 |
| GolayBarcode116 | ATCACTAGTCAC | 13_071 | 71 | 12 | E | ERP005251 | ERS421603 |
| GolayBarcode119 | ATCCTCAGTAGT | 13_072 | 72 | 12 | E | ERP007059 | ERS551400 |
| GolayBarcode124 | ATCTACTACACG | 13_074 | 74 | 12 | E | ERP005251 | ERS421603 |
| GolayBarcode132 | ATGATCGAGAGA | 13_077 | 77 | 12 | E | ERP005251 | ERS421603 |
| GolayBarcode140 | ATGGTCTACTAC | 13_079 | 79 | 12 | T | ERP005251 | ERS421603 |
| GolayBarcode147 | CAACACGCACGA | 13_081 | 81 | 12 | T | ERP005251 | ERS421603 |
| GolayBarcode476 | TAGCTGAGTCCA | 13_083 | 83 | 12 | T | ERP005251 | ERS421603 |
| GolayBarcode101 | AGTGTTCGATCG | 13_086 | 86 | 12 | T | ERP005251 | ERS421603 |
| GolayBarcode84 | AGCTCTCAGAGG | 13_090 | 90 | 12 | R | ERP007059 | ERS551400 |
| GolayBarcode109 | ATACTATTGCGC | 13_091 | 91 | 12 | R | ERP005251 | ERS421603 |
| GolayBarcode117 | ATCAGGCGTGTG | 13_094 | 94 | 12 | R | ERP005251 | ERS421603 |
| GolayBarcode43 | ACGTGCCGTAGA | 13_099 | 99 | 12 | B1 | ERP007059 | ERS551400 |

Table S1 continued.

| **Sanger Barcode ID** | **Barcode** | **Sputum ID** | **Sample No** | **Patient** | **Clinical Period** | **Study accession number** | **Sample accession number** |
| --- | --- | --- | --- | --- | --- | --- | --- |
| GolayBarcode456 | TACGATGACCAC | 13_106 | 106 | 12 | B1 | ERP007059 | ERS551400 |
| GolayBarcode140 | ATGGTCTACTAC | 13_113 | 113 | 12 | B1 | ERP007059 | ERS551400 |
| GolayBarcode23 | ACATTCAGCGCA | 13_142 | 142 | 12 | B1 | ERP007059 | ERS551400 |
| GolayBarcode11 | ACACGGTGTCTA | 13_149 | 149 | 12 | B1 | ERP007059 | ERS551400 |
| GolayBarcode480 | TAGTGTGCTTCA | Negative Control | |  |  | ERP005251 | ERS421603 |
| GolayBarcode420 | GTCGACTCCTCT | Negative Control | |  |  | ERP007059 | ERS551400 |
