# Supplementary material for: Respiratory microbiota resistance and resilience to pulmonary exacerbation and subsequent antimicrobial intervention
Source: ISME J. 2015 Nov 10;10(5):1081–91. doi: 10.1038/ismej.2015.198 (PMC4820042; doi:10.1038/ismej.2015.198)
Supplement: Supplementary Table S2 [file ismej2015198x3.doc]

**Table S2** Bacterial OTUs detected across samples from all patients and disease periods (*n* = 237). Ae and An denote aerobe and anaerobe, respectively. Only strict anaerobes were classified as anaerobes, whereas aerobes, facultative anaerobes, and microaerophiles were classified as aerobes. Species level identities of detected taxa are reported here. However, given the relatively short length of the ribosomal sequences analysed, these identities should be considered putative as it is often not possible to assign a single species identity to OTUs as more than one species can have identical matches.

| **Class** | **Family** | **Taxon name** | **Common / Rare** | **Anaerobe/ Aerobe** |
| --- | --- | --- | --- | --- |
| Actinobacteria | Actinomycetaceae | *Actinobaculum massiliense* | Rare | Ae |
|  |  | *Actinomyces graevenitzii* | Rare | Ae |
|  |  | *Actinomyces naeslundii* | Rare | Ae |
|  |  | *Actinomyces odontolyticus* | Rare | Ae |
|  | Bifidobacteriaceae | *Scardovia inopinata* | Rare | Ae |
|  | Coriobacteriaceae | *Atopobium parvulum* | Rare | An |
|  | Corynebacteriaceae | *Corynebacterium durum* | Rare | Ae |
|  |  | *Corynebacterium matruchotii* | Rare | Ae |
|  |  | *Corynebacterium tuberculostearicum* | Rare | Ae |
|  | Microbacteriaceae | *Microbacterium paraoxydans* | Rare | Ae |
|  | Micrococcaceae | *Arthrobacter tumbae* | Rare | An |
|  |  | *Rothia mucilaginosa* | Rare | Ae |
|  | Nocardiaceae | *Nocardia cyriacigeorgica* | Rare | An |
|  | Propionibacteriaceae | *Propionibacterium acidifaciens* | Rare | An |
|  |  | *Propionibacterium acnes* | Rare | An |
|  |  | *Propionibacterium propionicum* | Rare | Ae |
| Alphaproteobacteria | Brucellaceae | *Ochrobactrum anthropi* | Rare | Ae |
|  | Caulobacteraceae | *Brevundimonas vesicularis* | Rare | Ae |
|  | Rhodobacteraceae | *Paracoccus yeei* | Rare | Ae |
|  | Sphingomonadaceae | *Sphingobium amiense* | Rare | An |
|  |  | *Sphingomonas paucimobilis* | Rare | An |
| Bacilli | Aerococcaceae | *Abiotrophia defectiva* | Rare | Ae |
|  | Bacillaceae | *Bacillus subtilis* | Rare | Ae |
|  |  | *Gemella sanguinis* | Rare | Ae |
|  | Carnobacteriaceae | *Granulicatella adiacens* | Rare | Ae |
|  | Lactobacillaceae | *Lactobacillus casei* | Rare | Ae |
|  |  | *Lactobacillus delbrueckii* | Rare | Ae |
|  |  | *Lactobacillus frumenti* | Rare | Ae |
|  |  | *Lactobacillus johnsonii* | Rare | Ae |
|  |  | *Lactobacillus salivarius* | Rare | Ae |
|  | Staphylococcaceae | *Staphylococcus aureus* | Rare | Ae |
|  | Streptococcaceae | *Streptococcus agalactiae* | Rare | An |
|  |  | *Streptococcus mutans* | Rare | An |
|  |  | *Streptococcus pneumoniae* group | Common | An |
|  |  | *Streptococcus sanguinis* group | Common | An |
| Bacteroidia | Bacteroidaceae | *Bacteroides acidofaciens* | Rare | An |
|  |  | *Bacteroides cellulosilyticus* | Rare | An |
|  |  | *Bacteroides intestinalis* | Rare | An |
|  |  | *Bacteroides oleiciplenus* | Rare | An |
|  |  | *Bacteroides uniformis* | Rare | An |
|  | Porphyromonadaceae | *Barnesiella intestinihominis* | Rare | An |
|  |  | *Odoribacter laneus* | Rare | an |
| Table S2 continued |  |  |  |  |
| **Class** | **Family** | **Taxon name** | **Common / Rare** | **Anaerobe/ Aerobe** |
|  |  | *Paludibacter propionicigenes* | Rare | An |
|  |  | *Parabacteroides distasonis* | Rare | Ae |
|  |  | *Parabacteroides goldsteinii* | Rare | Ae |
|  |  | *Porphyromonas catoniae* | Rare | An |
|  |  | *Porphyromonas endodontalis* | Rare | An |
|  |  | *Porphyromonas somerae* | Rare | An |
|  |  | *Tannerella forsythia* | Rare | An |
|  | Prevotellaceae | *Prevotella bivia* | Rare | An |
|  |  | *Prevotella buccae* | Rare | An |
|  |  | *Prevotella copri* | Rare | An |
|  |  | *Prevotella denticola* | Rare | An |
|  |  | *Prevotella enoeca* | Rare | An |
|  |  | *Prevotella histicola* | Rare | An |
|  |  | *Prevotella loescheii* | Rare | An |
|  |  | *Prevotella maculosa* | Rare | An |
|  |  | *Prevotella melaninogenica* | Common | An |
|  |  | *Prevotella nanceiensis* | Rare | An |
|  |  | *Prevotella nigrescens* | Rare | An |
|  |  | *Prevotella oralis* | Rare | An |
|  |  | *Prevotella oris* | Rare | An |
|  |  | *Prevotella oulorum* | Rare | An |
|  |  | *Prevotella pallens* | Rare | An |
|  |  | *Prevotella paludivivens* | Rare | An |
|  |  | *Prevotella shahii* | Rare | An |
|  |  | *Prevotella tannerae* | Rare | An |
|  | Rikenellaceae | *Alistipes finegoldii* | Rare | An |
| Betaproteobacteria | Alcaligenaceae | *Achromobacter xylosoxidans* | Rare | Ae |
|  |  | *Advenella mimigardefordensis* | Rare | Ae |
|  | Burkholderiaceae | *Burkholderia cepacia complex* | Rare | Ae |
|  |  | *Lautropia mirabilis* | Rare | Ae |
|  |  | *Ralstonia mannitolilytica* | Rare | Ae |
|  | Burkholderiales *incertae sedis* | *Aquabacterium fontiphilum* | Rare | Ae |
|  | Comamonadaceae | *Comamonas testosteroni* | Rare | Ae |
|  |  | *Curvibacter lanceolatus* | Rare | Ae |
|  |  | *Delftia acidovorans* | Rare | Ae |
|  | Neisseriaceae | *Kingella oralis* | Rare | Ae |
|  |  | *Neisseria mucosa* | Rare | Ae |
|  |  | *Neisseria oralis* | Rare | Ae |
|  | Sutterellaceae | *Parasutterella excrementihominis* | Rare | An |
| Clostridia | Clostridiaceae | *Anaerococcus octavius* | Rare | An |
|  |  | *Clostridium aerotolerans* | Rare | An |
|  |  | *Clostridium aldenense* | Rare | An |
|  |  | *Clostridium algidixylanolyticum* | Rare | An |
|  |  | *Clostridium bolteae* | Rare | An |
|  |  | *Clostridium celerecrescens* | Rare | An |
|  |  | *Clostridium cellobioparum* | Rare | An |
| Table S2 continued |  |  |  |  |
| **Class** | **Family** | **Taxon name** | **Common / Rare** | **Anaerobe/ Aerobe** |
|  |  | *Clostridium clostridioforme* | Rare | An |
|  |  | *Clostridium hathewayi* | Rare | An |
|  |  | *Clostridium indolis* | Rare | An |
|  |  | *Clostridium lavalense* | Rare | An |
|  |  | *Clostridium orbiscindens* | Rare | An |
|  |  | *Clostridium papyrosolvens* | Rare |  |
|  |  | *Clostridium piliforme* | Rare | An |
|  |  | *Clostridium populeti* | Rare | An |
|  |  | *Clostridium proteoclasticum* | Rare | An |
|  |  | *Clostridium scindens* | Rare | An |
|  |  | *Clostridium subterminale* | Rare | An |
|  |  | *Clostridium sufflavum* | Rare | An |
|  |  | *Clostridium tertium* | Rare | An |
|  |  | *Finegoldia magna* | Rare | An |
|  |  | *Mogibacterium neglectum* | Rare | An |
|  |  | *Parvimonas micros* | Rare | An |
|  |  | *Peptoniphilus lacrimalis* | Rare | An |
|  | Eubacteriaceae | *Eubacterium brachy* | Rare | An |
|  |  | *Eubacterium sulci* | Rare | An |
|  |  | *Eubacterium ventriosum* | Rare | An |
|  | Lachnospiraceae | *Anaerostipes butyraticus* | Rare | An |
|  |  | *Blautia obeum* | Rare | An |
|  |  | *Catonella morbi* | Rare | An |
|  |  | *Howardella ureilytica* | Rare | An |
|  |  | *Lachnoanaerobaculum orale* | Rare | An |
|  |  | *Oribacterium sinus* | Rare | An |
|  |  | *Robinsoniella peoriensis* | Rare | An |
|  |  | *Shuttleworthia satelles* | Rare | An |
|  |  | *Stomatobaculum longum* | Rare | An |
|  | Peptococcaceae | *Peptococcus niger* | Rare | An |
|  | Peptostreptococcaceae | *Peptostreptococcus stomatis* | Rare | An |
|  | Ruminococcaceae | *Pseudoflavonifractor capillosus* | Rare | Ae |
|  |  | *Ruminococcus flavefaciens* | Rare | An |
| Deltaproteobacteria | Bdellovibrionaceae | *Vampirovibrio chlorellavorus* | Rare | Ae |
| Epsilonproteobacteria | Campylobacteraceae | *Campylobacter concisus* | Rare | Ae |
|  |  | *Campylobacter curvus* | Rare | Ae |
|  |  | *Campylobacter showae* | Rare | Ae |
| Erysipelotrichia | Erysipelotrichaceae | *Eubacterium cylindroides* | Rare | An |
| Flavobacteria | Flavobacteriaceae | *Capnocytophaga granulosa* | Rare | Ae |
|  |  | *Capnocytophaga ochracea* | Rare | Ae |
|  |  | *Capnocytophaga sputigena* | Rare | Ae |
|  |  | *Chryseobacterium indologenes* | Rare | Ae |
| Fusobacteria | Fusobacteriaceae | *Fusobacterium necrophorum* | Rare | An |
|  |  | *Fusobacterium nucleatum* | Rare | An |
|  | Leptotrichiaceae | *Leptotrichia buccalis* | Rare | Ae |
|  |  | *Sneathia sanguinegens* | Rare | Ae |
| Table S2 continued |  |  |  |  |
| **Class** | **Family** | **Taxon name** | **Common / Rare** | **Anaerobe/ Aerobe** |
| Gammaproteobacteria | Cardiobacteriaceae | *Cardiobacterium valvarum* | Rare | Ae |
|  | Enterobacteriaceae | *Enterobacter cowanii* | Rare | Ae |
|  |  | *Proteus mirabilis* | Rare | Ae |
|  |  | *Yersinia frederiksenii* | Rare | Ae |
|  | Moraxellaceae | *Acinetobacter johnsonii* | Rare | Ae |
|  |  | *Acinetobacter lwoffii* | Rare | Ae |
|  |  | *Moraxella nonliquefaciens* | Rare | Ae |
|  |  | *Moraxella osloensis* | Rare | Ae |
|  | Pasteurellaceae | *Haemophilus influenzae* | Rare | Ae |
|  |  | *Haemophilus parainfluenzae* | Rare | Ae |
|  | Pseudomonadaceae | *Pseudomonas aeruginosa* | Common | Ae |
|  |  | *Pseudomonas fragi* | Rare | Ae |
|  |  | *Pseudomonas pseudoalcaligenes* | Rare | Ae |
|  | Xanthomonadaceae | *Stenotrophomonas maltophilia* | Rare | An |
| Mollicutes | Mycoplasmataceae | *Mycoplasma hominis* | Rare | Ae |
|  |  | *Mycoplasma salivarium* | Rare | An |
| Negativicutes | Veillonellaceae | *Anaeroglobus geminatus* | Rare | An |
|  |  | *Dialister invisus* | Rare | Ae |
|  |  | *Dialister micraerophilus* | Rare | Ae |
|  |  | *Dialister pneumosintes* | Rare | Ae |
|  |  | *Megasphaera micronuciformis* | Rare | An |
|  |  | *Schwartzia succinivorans* | Rare | An |
|  |  | *Selenomonas artemidis* | Rare | An |
|  |  | *Selenomonas noxia* | Rare | An |
|  |  | *Veillonella parvula* | Common | An |
|  |  | *Veillonella ratti* | Rare | An |
| Sphingobacteria | Chitinophagaceae | *Sediminibacterium salmoneum* | Rare | Ae |
|  | Sphingobacteriaceae | *Sphingobacterium spiritivorum* | Rare | Ae |
| Spirochaetes | Spirochaetaceae | *Treponema denticola* | Rare | Ae |
