# Supplementary material for: Respiratory microbiota resistance and resilience to pulmonary exacerbation and subsequent antimicrobial intervention
Source: ISME J. 2015 Nov 10;10(5):1081–91. doi: 10.1038/ismej.2015.198 (PMC4820042; doi:10.1038/ismej.2015.198)
Supplement: Supplementary Table S3 [file ismej2015198x4.doc]

**Table S3** Average numbers of bacterial sequence reads across all samples and within the five disease states. Given is the mean number of bacterial sequence reads, the standard deviation of the mean (SD) and maximum and minimum number of reads, in each instance.

|  | *n* | Mean | SD | Max | Min |
| --- | --- | --- | --- | --- | --- |
| All | 237 | 1628.7 | 83.7 | 9123 | 602 |
| B_0_ | 56 | 1817.4 | 244.4 | 9123 | 617 |
| E | 41 | 1357.5 | 134.7 | 3476 | 695 |
| T | 67 | 1643.5 | 126.4 | 4344 | 617 |
| R | 32 | 1845.1 | 148.4 | 3985 | 606 |
| B_1_ | 41 | 1449.0 | 209.1 | 5621 | 602 |
