# Supplementary material for: Respiratory microbiota resistance and resilience to pulmonary exacerbation and subsequent antimicrobial intervention
Source: ISME J. 2015 Nov 10;10(5):1081–91. doi: 10.1038/ismej.2015.198 (PMC4820042; doi:10.1038/ismej.2015.198)
Supplement: Supplementary Table S4 [file ismej2015198x5.doc]

**Table S4** Similarity of percentages (SIMPER) analysis of bacterial dissimilarity (Bray-Curtis) of the whole microbiota between disease states. Given is mean % abundance of sequences for each species across the periods they were observed to occupy. Also given is average dissimilarity and percentage contribution, calculated from the mean contribution divided by mean dissimilarity between periods. (a) SIMPER analysis of whole microbiota between disease periods B_0_ and E, (b) SIMPER analysis between periods E and T, (c) periods T and R, (d) R and B_1_, and (e) B_0_ and B_1_. Asterisks denote species belonging to the core OTU group and species names highlighted in bold are considered to be recognised CF pathogens.

| **A** |  |  |  |  |  |  |
| --- | --- | --- | --- | --- | --- | --- |
|  | **Taxon** | **% Mean abundance** | | **Average dissimilarity** | **Contribution %** | **Cumulative %** |
|  |  | **B_0_** | **E** |  |  |  |
|  | ***Pseudomonas aeruginosa**** | 53.9 | 49 | 22.03 | 37.44 | 37.44 |
|  | ***Streptococcus pneumoniae* group***** | 8.22 | 12.9 | 7.056 | 11.99 | 49.42 |
|  | *Streptococcus sanguinis* group* | 8.53 | 9.5 | 5.603 | 9.521 | 58.95 |
|  | *Prevotella melaninogenica** | 9.35 | 5.12 | 5.128 | 8.714 | 67.66 |
|  | *Porphyromonas catoniae* | 4.91 | 2.99 | 3.142 | 5.339 | 73 |
|  | *Veillonella parvula** | 1.21 | 5.41 | 2.605 | 4.426 | 77.42 |
|  | *Fusobacterium nucleatum* | 2.03 | 2.76 | 2.215 | 3.763 | 81.19 |
|  | *Enterobacter cowanii* | 0.0144 | 2.51 | 1.262 | 2.144 | 83.33 |
|  | ***Stenotrophomonas maltophilia*** | 2.37 | 0.141 | 1.225 | 2.082 | 85.41 |
|  | *Prevotella enoeca* | 0.559 | 0.915 | 0.7029 | 1.194 | 86.61 |
|  | *Prevotella oris* | 1.22 | 0.315 | 0.6859 | 1.165 | 87.77 |
|  | ***Staphylococcus aureus*** | 0.0172 | 1.13 | 0.5699 | 0.9683 | 88.74 |
|  | *Bacteroides oleiciplenus* | 0.784 | 0.546 | 0.5613 | 0.9537 | 89.7 |
|  | *Megasphaera micronuciformis* | 0.729 | 0.352 | 0.4751 | 0.8072 | 90.5 |

| **B** |  |  |  |  |  |  |
| --- | --- | --- | --- | --- | --- | --- |
|  | **Taxon** | **% Mean abundance** | | **Average dissimilarity** | **Contribution %** | **Cumulative %** |
|  |  | **E** | **T** |  |  |  |
|  | ***Pseudomonas aeruginosa**** | 49 | 57.2 | 21.81 | 37.33 | 37.33 |
|  | ***Streptococcus pneumoniae* group***** | 12.9 | 12 | 8.164 | 13.97 | 51.3 |
|  | *Streptococcus sanguinis* group* | 9.5 | 3.37 | 4.57 | 7.823 | 59.13 |
|  | *Prevotella melaninogenica** | 5.12 | 4.5 | 3.452 | 5.91 | 65.04 |
|  | *Veillonella parvula** | 5.41 | 4.07 | 3.387 | 5.798 | 70.84 |
|  | *Enterobacter cowanii* | 2.51 | 3.95 | 3.061 | 5.239 | 76.07 |
|  | *Fusobacterium nucleatum* | 2.76 | 2.09 | 2.259 | 3.867 | 79.94 |
|  | *Porphyromonas catoniae* | 2.99 | 0.89 | 1.726 | 2.954 | 82.9 |
|  | ***Staphylococcus aureus*** | 1.13 | 1.66 | 1.339 | 2.293 | 85.19 |
|  | *Prevotella enoeca* | 0.915 | 1.04 | 0.9239 | 1.582 | 86.77 |
|  | *Achromobacter xylosoxidans* | 0.19 | 1.03 | 0.5872 | 1.005 | 87.77 |
|  | *Haemophilus parainfluenzae* | 0.381 | 0.828 | 0.546 | 0.9346 | 88.71 |
|  | *Barnesiella intestinihominis* | 0.269 | 0.844 | 0.5298 | 0.9069 | 89.62 |
|  | ***Stenotrophomonas maltophilia*** | 0.141 | 0.904 | 0.5051 | 0.8647 | 90.48 |

Table S3 continued

| **C** |  |  |  |  |  |  |
| --- | --- | --- | --- | --- | --- | --- |
|  | **Taxon** | **% Mean abundance** | | **Average dissimilarity** | **Contribution %** | **Cumulative %** |
|  |  | **T** | **R** |  |  |  |
|  | ***Pseudomonas aeruginosa**** | 57.2 | 61.9 | 21.2 | 38.84 | 38.84 |
|  | ***Streptococcus pneumoniae* group***** | 12 | 9.4 | 7.706 | 14.12 | 52.96 |
|  | *Fusobacterium nucleatum* | 2.09 | 6.92 | 4.206 | 7.706 | 60.67 |
|  | *Prevotella melaninogenica** | 4.5 | 3.53 | 3.247 | 5.949 | 66.62 |
|  | *Veillonella parvula** | 4.07 | 4.2 | 2.974 | 5.45 | 72.07 |
|  | *Streptococcus sanguinis* group* | 3.37 | 3.37 | 2.319 | 4.249 | 76.32 |
|  | *Enterobacter cowanii* | 3.95 | 0.0414 | 1.987 | 3.64 | 79.96 |
|  | *Porphyromonas catoniae* | 0.89 | 3.15 | 1.842 | 3.374 | 83.33 |
|  | *Prevotella enoeca* | 1.04 | 1.84 | 1.323 | 2.424 | 85.76 |
|  | ***Staphylococcus aureus*** | 1.66 | 0.0042 | 0.8324 | 1.525 | 87.28 |
|  | ***Achromobacter xylosoxidans*** | 1.03 | 0.309 | 0.6343 | 1.162 | 88.45 |
|  | ***Stenotrophomonas maltophilia*** | 0.904 | 0.0233 | 0.4585 | 0.8402 | 89.29 |
|  | *Haemophilus parainfluenzae* | 0.828 | 0.116 | 0.449 | 0.8227 | 90.11 |
|  | *Barnesiella intestinihominis* | 0.844 | 0.0338 | 0.4308 | 0.7894 | 90.9 |

| **D** |  |  |  |  |  |  |
| --- | --- | --- | --- | --- | --- | --- |
|  | **Taxon** | **% Mean abundance** | | **Average dissimilarity** | **Contribution %** | **Cumulative %** |
|  |  | **R** | **B_1_** |  |  |  |
|  | ***Pseudomonas aeruginosa**** | 61.9 | 53.5 | 20.4 | 37.42 | 37.42 |
|  | ***Streptococcus pneumoniae* group***** | 9.4 | 9.45 | 6.589 | 12.08 | 49.5 |
|  | *Fusobacterium nucleatum* | 6.92 | 3.94 | 4.838 | 8.872 | 58.38 |
|  | *Streptococcus sanguinis* group* | 3.37 | 9.44 | 4.448 | 8.157 | 66.53 |
|  | *Prevotella melaninogenica** | 3.53 | 4.78 | 3.238 | 5.939 | 72.47 |
|  | *Porphyromonas catoniae* | 3.15 | 2 | 2.154 | 3.951 | 76.42 |
|  | *Veillonella parvula** | 4.2 | 1.3 | 2.133 | 3.912 | 80.34 |
|  | *Prevotella enoeca* | 1.84 | 1.69 | 1.539 | 2.823 | 83.16 |
|  | ***Stenotrophomonas maltophilia*** | 0.0233 | 1.79 | 0.8973 | 1.646 | 84.8 |
|  | ***Staphylococcus aureus*** | 0.0042 | 1.76 | 0.8823 | 1.618 | 86.42 |
|  | *Neisseria mucosa* | 0.155 | 1.44 | 0.766 | 1.405 | 87.83 |
|  | *Nocardia cyriacigeorgica* | 0.00775 | 1.27 | 0.6387 | 1.171 | 89 |
|  | *Prevotella oris* | 0.598 | 0.742 | 0.5766 | 1.058 | 90.06 |
|  | *Sneathia sanguinegens* | 0.706 | 0.49 | 0.5459 | 1.001 | 91.06 |

Table S3 continued

| **E** |  |  |  |  |  |  |
| --- | --- | --- | --- | --- | --- | --- |
|  | **Taxon** | **% Mean abundance** | | **Average dissimilarity** | **Contribution %** | **Cumulative %** |
|  |  | **B_0_** | **B_1_** |  |  |  |
|  | ***Pseudomonas aeruginosa**** | 53.9 | 53.5 | 21.43 | 37.7 | 37.7 |
|  | ***Streptococcus pneumoniae* group***** | 8.22 | 9.45 | 5.889 | 10.36 | 48.06 |
|  | *Streptococcus sanguinis* group* | 8.53 | 9.44 | 5.508 | 9.689 | 57.75 |
|  | *Prevotella melaninogenica** | 9.35 | 4.78 | 5.048 | 8.879 | 66.63 |
|  | *Porphyromonas catoniae* | 4.91 | 2 | 2.72 | 4.785 | 71.41 |
|  | *Fusobacterium nucleatum* | 2.03 | 3.94 | 2.717 | 4.779 | 76.19 |
|  | ***Stenotrophomonas maltophilia*** | 2.37 | 1.79 | 1.953 | 3.436 | 79.63 |
|  | *Prevotella enoeca* | 0.559 | 1.69 | 1.049 | 1.846 | 81.47 |
|  | *Neisseria mucosa* | 0.717 | 1.44 | 1.004 | 1.766 | 83.24 |
|  | *Veillonella parvula** | 1.21 | 1.3 | 0.8938 | 1.572 | 84.81 |
|  | ***Staphylococcus aureus*** | 0.0172 | 1.76 | 0.8881 | 1.562 | 86.37 |
|  | *Prevotella oris* | 1.22 | 0.742 | 0.8703 | 1.531 | 87.91 |
|  | *Nocardia cyriacigeorgica* | 0.00321 | 1.27 | 0.6369 | 1.12 | 89.03 |
|  | *Parvimonas micros* | 0.333 | 0.685 | 0.4852 | 0.8535 | 89.88 |
|  | *Bacteroides oleiciplenus* | 0.784 | 0.321 | 0.476 | 0.8373 | 90.72 |
