# Supplementary material for: Respiratory microbiota resistance and resilience to pulmonary exacerbation and subsequent antimicrobial intervention
Source: ISME J. 2015 Nov 10;10(5):1081–91. doi: 10.1038/ismej.2015.198 (PMC4820042; doi:10.1038/ismej.2015.198)
Supplement: Supplementary Table S5 [file ismej2015198x6.doc]

**Table S5** Similarity of percentages (SIMPER) analysis of bacterial dissimilarity (Bray-Curtis) of the common OTU group between disease states. Given is mean % abundance of sequences for each species across the periods they were observed to occupy. Also given is average dissimilarity and percentage contribution, calculated from the mean contribution divided by mean dissimilarity between periods. (a) SIMPER analysis of common microbiota between disease periods B_0_ and E, (b) SIMPER analysis between periods E and T, (c) periods T and R, (d) R and B_1_, and (e) B_0_ and B_1_. Species names highlighted in bold are considered to be recognised CF pathogens.

| **A** |  | |  | |  | |  | |  | | |  | | |
| --- | --- | --- | --- | --- | --- | --- | --- | --- | --- | --- | --- | --- | --- | --- |
|  | ***Taxon*** | | **% Mean abundance** | | | | **Average dissimilarity** | | **Contribution %** | | | **Cumulative %** | | |
|  |  |  | **B_0_** | | **E** | |  |  |  |  |  |  |  |  |
|  | ***Pseudomonas aeruginosa*** | | 53.9 | | 49 | | 27.55 | | 51.44 | | | 51.44 | | |
|  | ***Streptococcus pneumoniae* group** | | 8.22 | | 12.9 | | 8.933 | | 16.68 | | | 68.11 | | |
|  | *Streptococcus sanguinis* group | | 8.53 | | 9.5 | | 7.075 | | 13.21 | | | 81.32 | | |
|  | *Prevotella melaninogenica* | | 9.35 | | 5.12 | | 6.701 | | 12.51 | | | 93.83 | | |
|  | *Veillonella parvula* | | 1.21 | | 5.41 | | 3.304 | | 6.167 | | | 100 | | |
| **B** |  | |  | |  | |  | |  | | |  | | |
|  | ***Taxon*** | **% Mean abundance** | | | | **Average dissimilarity** | | **Contribution %** | | | **Cumulative %** | | |  |
|  |  | **E** | | **T** | |  |  |  |  |  |  |  |  |  |
|  | ***Pseudomonas aeruginosa*** | 49 | | 57.2 | | 29.8 | | 54.55 | | | 54.55 | | |  |
|  | ***Streptococcus pneumoniae* group** | 12.9 | | 12 | | 11.33 | | 19.54 | | | 75.23 | | |  |
|  | *Streptococcus sanguinis* group | 9.5 | | 3.37 | | 6.272 | | 11.45 | | | 86.68 | | |  |
|  | *Veillonella parvula* | 5.41 | | 0 | | 3.741 | | 6.828 | | | 93.51 | | |  |
|  | *Prevotella melaninogenica* | 5.12 | | 0 | | 3.555 | | 6.488 | | | 100 | | |  |
| **C** |  |  | |  | |  | |  | | |  | | |  |
|  | ***Taxon*** | **% Mean abundance** | | | | **Average dissimilarity** | | **Contribution %** | | | **Cumulative %** | | |  |
|  |  | **T** | | **R** | |  |  |  |  |  |  |  |  |  |
|  | ***Pseudomonas aeruginosa*** | 57.2 | | 61.9 | | 28.3 | | 61.11 | | | 61.11 | | |  |
|  | ***Streptococcus pneumoniae* group** | 12 | | 9.4 | | 9.852 | | 21.87 | | | 82.98 | | |  |
|  | *Streptococcus sanguinis* group | 3.37 | | 3.37 | | 3.03 | | 6.594 | | | 89.58 | | |  |
|  | *Veillonella parvula* | 0 | | 4.2 | | 2.836 | | 5.68 | | | 95.25 | | |  |
|  | *Prevotella melaninogenica* | 0 | | 3.53 | | 2.369 | | 4.745 | | | 100 | | |  |
| **D** |  |  | |  | |  | |  | | |  | | |  |
|  | ***Taxon*** | **% Mean abundance** | | | | **Average dissimilarity** | | **Contribution %** | | | **Cumulative %** | | |  |
|  |  | **R** | | **B_1_** | |  |  |  |  |  |  |  |  |  |
|  | ***Pseudomonas aeruginosa*** | 61.9 | | 53.5 | | 27.59 | | 56.28 | | | 56.28 | | |  |
|  | ***Streptococcus pneumoniae* group** | 9.4 | | 9.45 | | 8.492 | | 17.32 | | | 73.61 | | |  |
|  | *Streptococcus sanguinis* group | 3.37 | | 9.44 | | 5.907 | | 12.05 | | | 85.66 | | |  |
|  | *Prevotella melaninogenica* | 3.53 | | 4.78 | | 4.211 | | 8.59 | | | 94.25 | | |  |
|  | *Veillonella parvula* | 4.2 | | 1.3 | | 2.82 | | 5.753 | | | 100 | | |  |
| **E** |  | |  | |  | |  | |  |  | | |  | |
|  | ***Taxon*** | | **% Mean abundance** | | | | **Average dissimilarity** | | **Contribution %** | **Cumulative %** | | |  | |
|  |  |  | **B_0_** | | **B_1_** | |  |  |  |  |  |  |  | |
|  | ***Pseudomonas aeruginosa*** | | 53.9 | | 53.5 | | 28.16 | | 54.87 | 54.87 | | |  | |
|  | ***Streptococcus pneumoniae* group** | | 8.22 | | 9.45 | | 7.685 | | 14.97 | 69.84 | | |  | |
|  | *Streptococcus sanguinis* group | | 8.53 | | 9.44 | | 7.275 | | 14.17 | 84.02 | | |  | |
|  | *Prevotella melaninogenica* | | 9.35 | | 4.78 | | 6.892 | | 13.43 | 97.44 | | |  | |
|  | *Veillonella parvula* | | 1.21 | | 1.3 | | 1.311 | | 2.555 | 100 | | |  | |
