# Supplementary material for: Respiratory microbiota resistance and resilience to pulmonary exacerbation and subsequent antimicrobial intervention
Source: ISME J. 2015 Nov 10;10(5):1081–91. doi: 10.1038/ismej.2015.198 (PMC4820042; doi:10.1038/ismej.2015.198)
Supplement: Supplementary Table S6 [file ismej2015198x7.doc]

**Table S6** Similarity of percentages (SIMPER) analysis of bacterial dissimilarity (Bray-Curtis) of the rare OTU group between disease states. Given is mean % abundance of sequences for each species across the periods they were observed to occupy. Also given is average dissimilarity and percentage contribution, calculated from the mean contribution divided by mean dissimilarity between periods. (a) SIMPER analysis of rare microbiota between disease periods B_0_ and E, (b) SIMPER analysis between periods E and T, (c) periods T and R, (d) R and B_1_, and (e) B_0_ and B_1_. Species names highlighted in bold are considered to be recognised CF pathogens.

| **A** |  |  |  |  |  |  |
| --- | --- | --- | --- | --- | --- | --- |
|  | **Taxon** | **% Mean abundance** | | **Average dissimilarity** | **Contribution %** | **Cumulative %** |
|  |  | **B_1_** | **E** |  |  |  |
|  | *Porphyromonas catoniae* | 4.91 | 2.99 | 17.67 | 19.48 | 19.48 |
|  | *Fusobacterium nucleatum* | 2.03 | 2.76 | 6.669 | 7.352 | 26.84 |
|  | ***Stenotrophomonas maltophilia*** | 2.37 | 0.141 | 5.237 | 5.773 | 32.61 |
|  | *Enterobacter cowanii* | 0.0144 | 2.51 | 4.923 | 5.428 | 38.04 |
|  | *Prevotella oris* | 1.22 | 0.315 | 3.649 | 4.023 | 42.06 |
|  | *Bacteroides oleiciplenus* | 0.784 | 0.546 | 3.486 | 3.843 | 45.9 |
|  | *Megasphaera micronuciformis* | 0.729 | 0.352 | 3.429 | 3.78 | 49.68 |
|  | *Haemophilus parainfluenzae* | 0.0138 | 0.381 | 2.926 | 3.226 | 52.91 |
|  | *Gemella sanguinis* | 0.183 | 0.489 | 2.905 | 3.203 | 56.11 |
|  | *Capnocytophaga sputigena* | 0.123 | 0.506 | 2.786 | 3.071 | 59.18 |
|  | *Prevotella maculosa* | 0.74 | 0.255 | 2.584 | 2.849 | 62.03 |
|  | *Prevotella pallens* | 0.471 | 0.401 | 2.427 | 2.676 | 64.71 |
|  | ***Staphylococcus aureus*** | 0.0172 | 1.13 | 2.413 | 2.66 | 67.37 |
|  | *Prevotella nanceiensis* | 0.305 | 0.349 | 2.09 | 2.304 | 69.67 |
|  | *Neisseria mucosa* | 0.717 | 0.177 | 2.064 | 2.275 | 71.95 |
|  | *Granulicatella adiacens* | 0.308 | 0.199 | 2.049 | 2.259 | 74.21 |
|  | *Capnocytophaga granulosa* | 0.17 | 0.409 | 2.043 | 2.253 | 76.46 |
|  | *Prevotella enoeca* | 0.559 | 0.915 | 1.722 | 1.898 | 78.36 |
|  | *Oribacterium sinus* | 0.257 | 0.139 | 1.593 | 1.757 | 80.12 |
|  | ***Achromobacter xylosoxidans*** | 0.028 | 0.19 | 1.323 | 1.459 | 81.57 |
|  | *Prevotella loescheii* | 0.283 | 0.123 | 1.251 | 1.379 | 82.95 |
|  | *Nocardia cyriacigeorgica* | 0.00321 | 0.181 | 1.243 | 1.37 | 84.32 |
|  | *Parvimonas micros* | 0.333 | 0.521 | 1.117 | 1.232 | 85.56 |
|  | *Porphyromonas endodontalis* | 0.416 | 0.0559 | 1.026 | 1.132 | 86.69 |
|  | *Lachnoanaerobaculum orale* | 0.218 | 0.149 | 0.9714 | 1.071 | 87.76 |
|  | *Actinomyces odontolyticus* | 0.282 | 0.0664 | 0.9427 | 1.039 | 88.8 |
|  | *Barnesiella intestinihominis* | 0.00679 | 0.269 | 0.7411 | 0.8171 | 89.62 |
|  | *Prevotella nigrescens* | 0.242 | 0.112 | 0.7262 | 0.8007 | 90.42 |

Table S5 continued

| **B** |  |  |  |  |  |  |
| --- | --- | --- | --- | --- | --- | --- |
|  | **Taxon** | **% Mean abundance** | | **Average dissimilarity** | **Contribution %** | **Cumulative %** |
|  |  | **E** | **T** |  |  |  |
|  | *Porphyromonas catoniae* | 0.89 | 2.99 | 9.571 | 10.11 | 10.11 |
|  | *Veillonella parvula* | 4.07 | 0 | 9.369 | 9.894 | 20 |
|  | *Prevotella melaninogenica* | 4.5 | 0 | 9.242 | 9.759 | 29.76 |
|  | *Enterobacter cowanii* | 3.95 | 2.51 | 8.502 | 8.978 | 38.74 |
|  | *Fusobacterium nucleatum* | 2.09 | 2.76 | 6.064 | 6.403 | 45.14 |
|  | ***Staphylococcus aureus*** | 1.66 | 1.13 | 3.873 | 4.09 | 49.23 |
|  | ***Achromobacter xylosoxidans*** | 1.03 | 0.19 | 3.609 | 3.811 | 53.04 |
|  | *Haemophilus parainfluenzae* | 0.828 | 0.381 | 3.414 | 3.605 | 56.65 |
|  | *Prevotella enoeca* | 1.04 | 0.915 | 2.764 | 2.919 | 59.57 |
|  | *Barnesiella intestinihominis* | 0.844 | 0.269 | 2.517 | 2.658 | 62.22 |
|  | *Capnocytophaga sputigena* | 0.0623 | 0.506 | 2.363 | 2.495 | 64.72 |
|  | *Gemella sanguinis* | 0.147 | 0.489 | 2.321 | 2.451 | 67.17 |
|  | *Prevotella pallens* | 0.559 | 0.401 | 2.13 | 2.249 | 69.42 |
|  | *Prevotella nanceiensis* | 0.404 | 0.349 | 2.025 | 2.138 | 71.56 |
|  | *Capnocytophaga granulosa* | 0.263 | 0.409 | 1.913 | 2.02 | 73.58 |
|  | *Bacteroides oleiciplenus* | 0.183 | 0.546 | 1.897 | 2.003 | 75.58 |
|  | *Megasphaera micronuciformis* | 0.196 | 0.352 | 1.816 | 1.917 | 77.5 |
|  | ***Stenotrophomonas maltophilia*** | 0.904 | 0.141 | 1.602 | 1.692 | 79.19 |
|  | *Prevotella oris* | 0.347 | 0.315 | 1.481 | 1.564 | 80.75 |
|  | *Prevotella maculosa* | 0.294 | 0.255 | 1.327 | 1.401 | 82.15 |
|  | *Neisseria mucosa* | 0.228 | 0.177 | 1.292 | 1.365 | 83.52 |
|  | *Granulicatella adiacens* | 0.0668 | 0.199 | 1.183 | 1.249 | 84.77 |
|  | *Nocardia cyriacigeorgica* | 0.00366 | 0.181 | 1.048 | 1.106 | 85.87 |
|  | *Oribacterium sinus* | 0.0538 | 0.139 | 0.9833 | 1.038 | 86.91 |
|  | *Lachnoanaerobaculum orale* | 0.252 | 0.149 | 0.8023 | 0.8472 | 87.76 |
|  | *Prevotella loescheii* | 0.2 | 0.123 | 0.7559 | 0.7982 | 88.56 |
|  | *Parvimonas micros* | 0.107 | 0.521 | 0.7398 | 0.7812 | 89.34 |
|  | *Prevotella nigrescens* | 0.114 | 0.112 | 0.5043 | 0.5326 | 89.87 |
|  | *Clostridium lavalense* | 0.125 | 0.066 | 0.4203 | 0.4438 | 90.32 |
|  | ***Haemophilus influenzae*** | 0.00545 | 0.0569 | 0.4048 | 0.4274 | 90.74 |

Tables S5 continued

| **C** |  |  |  |  |  |  |
| --- | --- | --- | --- | --- | --- | --- |
|  | **Taxon** | **% Mean abundance** | | **Average dissimilarity** | **Contribution %** | **Cumulative %** |
|  |  | **T** | **R** |  |  |  |
|  | *Veillonella parvula* | 0 | 4.07 | 11.62 | 12.15 | 12.15 |
|  | *Fusobacterium nucleatum* | 6.92 | 2.09 | 10.55 | 11.03 | 23.17 |
|  | *Prevotella melaninogenica* | 0 | 4.5 | 10.52 | 10.99 | 34.17 |
|  | *Porphyromonas catoniae* | 3.15 | 0.89 | 9.178 | 9.591 | 43.76 |
|  | *Enterobacter cowanii* | 0.0414 | 3.95 | 5.018 | 5.244 | 49 |
|  | ***Achromobacter xylosoxidans*** | 0.309 | 1.03 | 4.949 | 5.172 | 54.17 |
|  | *Prevotella enoeca* | 1.84 | 1.04 | 4.077 | 4.261 | 58.43 |
|  | *Haemophilus parainfluenzae* | 0.116 | 0.828 | 2.907 | 3.038 | 61.47 |
|  | *Barnesiella intestinihominis* | 0.0338 | 0.844 | 2.808 | 2.935 | 64.4 |
|  | *Capnocytophaga granulosa* | 0.336 | 0.263 | 2.131 | 2.227 | 66.63 |
|  | ***Staphylococcus aureus*** | 0.0042 | 1.66 | 2.02 | 2.111 | 68.74 |
|  | *Capnocytophaga sputigena* | 0.518 | 0.0623 | 1.864 | 1.948 | 70.69 |
|  | *Prevotella oris* | 0.598 | 0.347 | 1.736 | 1.814 | 72.5 |
|  | *Prevotella nanceiensis* | 0.167 | 0.404 | 1.7 | 1.777 | 74.28 |
|  | *Gemella sanguinis* | 0.248 | 0.147 | 1.667 | 1.742 | 76.02 |
|  | *Prevotella maculosa* | 0.242 | 0.294 | 1.622 | 1.695 | 77.72 |
|  | *Prevotella pallens* | 0.122 | 0.559 | 1.612 | 1.685 | 79.4 |
|  | *Neisseria mucosa* | 0.155 | 0.228 | 1.55 | 1.62 | 81.02 |
|  | ***Stenotrophomonas maltophilia*** | 0.0233 | 0.904 | 1.381 | 1.443 | 82.47 |
|  | *Megasphaera micronuciformis* | 0.156 | 0.196 | 1.195 | 1.249 | 83.72 |
|  | *Granulicatella adiacens* | 0.131 | 0.0668 | 1.078 | 1.127 | 84.84 |
|  | *Lachnoanaerobaculum orale* | 0.127 | 0.252 | 1.038 | 1.084 | 85.93 |
|  | *Sneathia sanguinegens* | 0.706 | 0.163 | 1.015 | 1.061 | 86.99 |
|  | *Bacteroides oleiciplenus* | 0.241 | 0.183 | 1.007 | 1.052 | 88.04 |
|  | *Prevotella loescheii* | 0.0527 | 0.2 | 0.756 | 0.79 | 88.83 |
|  | *Prevotella nigrescens* | 0.154 | 0.114 | 0.6753 | 0.7057 | 89.54 |
|  | *Parvimonas micros* | 0.295 | 0.107 | 0.4477 | 0.4678 | 90 |
|  | *Oribacterium sinus* | 0.0814 | 0.0538 | 0.4429 | 0.4628 | 90.47 |
|  | *Actinomyces odontolyticus* | 0.079 | 0.0785 | 0.4234 | 0.4425 | 90.91 |
|  | ***Haemophilus influenzae*** | 0.0212 | 0.00545 | 0.4034 | 0.4215 | 91.33 |
|  | *Atopobium parvulum* | 0.0531 | 0.0537 | 0.3991 | 0.4171 | 91.75 |
|  | *Clostridium lavalense* | 0 | 0.125 | 0.3509 | 0.3667 | 92.11 |
|  | *Prevotella oralis* | 0.0829 | 0.137 | 0.3405 | 0.3558 | 92.47 |

Tables S5 continued

| **D** |  |  |  |  |  |  |
| --- | --- | --- | --- | --- | --- | --- |
|  | **Taxon** | **% Mean abundance** | | **Average dissimilarity** | **Contribution %** | **Cumulative %** |
|  |  | **R** | **B_1_** |  |  |  |
|  | *Porphyromonas catoniae* | 3.15 | 2 | 14.6 | 15.95 | 15.95 |
|  | *Fusobacterium nucleatum* | 6.92 | 3.94 | 13.11 | 14.32 | 30.27 |
|  | *Prevotella enoeca* | 1.84 | 1.69 | 4.462 | 4.873 | 35.14 |
|  | ***Achromobacter xylosoxidans*** | 0.309 | 0.338 | 4.239 | 4.63 | 39.77 |
|  | *Nocardia cyriacigeorgica* | 0.00775 | 1.27 | 4.105 | 4.483 | 44.25 |
|  | *Granulicatella adiacens* | 0.131 | 0.446 | 3.765 | 4.112 | 48.37 |
|  | ***Stenotrophomonas maltophilia*** | 0.0233 | 1.79 | 3.392 | 3.704 | 52.07 |
|  | *Gemella sanguinis* | 0.248 | 0.54 | 3.378 | 3.689 | 55.76 |
|  | *Prevotella oris* | 0.598 | 0.742 | 3.094 | 3.379 | 59.14 |
|  | *Neisseria mucosa* | 0.155 | 1.44 | 3.035 | 3.315 | 62.45 |
|  | *Oribacterium sinus* | 0.0814 | 0.358 | 2.458 | 2.685 | 65.14 |
|  | ***Staphylococcus aureus*** | 0.0042 | 1.76 | 2.185 | 2.387 | 67.53 |
|  | *Bacteroides oleiciplenus* | 0.241 | 0.321 | 2.16 | 2.359 | 69.88 |
|  | *Capnocytophaga sputigena* | 0.518 | 0.0937 | 2.059 | 2.249 | 72.13 |
|  | *Prevotella maculosa* | 0.242 | 0.225 | 1.97 | 2.152 | 74.29 |
|  | *Capnocytophaga granulosa* | 0.336 | 0.0911 | 1.794 | 1.959 | 76.24 |
|  | *Haemophilus parainfluenzae* | 0.116 | 0.295 | 1.669 | 1.822 | 78.07 |
|  | *Actinomyces odontolyticus* | 0.079 | 0.247 | 1.662 | 1.815 | 79.88 |
|  | *Sneathia sanguinegens* | 0.706 | 0.49 | 1.506 | 1.644 | 81.53 |
|  | *Prevotella nanceiensis* | 0.167 | 0.221 | 1.381 | 1.508 | 83.04 |
|  | *Megasphaera micronuciformis* | 0.156 | 0.238 | 1.378 | 1.505 | 84.54 |
|  | *Parvimonas micros* | 0.295 | 0.685 | 1.197 | 1.307 | 85.85 |
|  | *Atopobium parvulum* | 0.0531 | 0.102 | 1.119 | 1.222 | 87.07 |
|  | *Prevotella nigrescens* | 0.154 | 0.493 | 1.086 | 1.186 | 88.26 |
|  | *Prevotella denticola* | 0.0059 | 0.135 | 0.9884 | 1.08 | 89.33 |
|  | *Lachnoanaerobaculum orale* | 0.127 | 0.122 | 0.9742 | 1.064 | 90.4 |

Tables S5 continued

| **E** |  |  |  |  |  |  |
| --- | --- | --- | --- | --- | --- | --- |
|  | **Taxon** | **% Mean abundance** | | **Average dissimilarity** | **Contribution %** | **Cumulative %** |
|  |  | **B_0_** | **B_1_** |  |  |  |
|  | *Porphyromonas catoniae* | 4.91 | 2 | 16.61 | 18.35 | 18.35 |
|  | *Fusobacterium nucleatum* | 2.03 | 3.94 | 8.157 | 9.008 | 27.36 |
|  | ***Stenotrophomonas maltophilia*** | 2.37 | 1.79 | 7.242 | 7.998 | 35.35 |
|  | *Prevotella oris* | 1.22 | 0.742 | 4.451 | 4.915 | 40.27 |
|  | *Granulicatella adiacens* | 0.308 | 0.446 | 3.744 | 4.135 | 44.4 |
|  | *Nocardia cyriacigeorgica* | 0.00321 | 1.27 | 3.696 | 4.082 | 48.49 |
|  | *Neisseria mucosa* | 0.717 | 1.44 | 3.25 | 3.589 | 52.07 |
|  | *Bacteroides oleiciplenus* | 0.784 | 0.321 | 3.107 | 3.431 | 55.51 |
|  | *Gemella sanguinis* | 0.183 | 0.54 | 3.045 | 3.362 | 58.87 |
|  | *Prevotella enoeca* | 0.559 | 1.69 | 3.042 | 3.36 | 62.23 |
|  | ***Achromobacter xylosoxidans*** | 0.028 | 0.338 | 2.668 | 2.946 | 65.17 |
|  | *Prevotella maculosa* | 0.74 | 0.225 | 2.551 | 2.818 | 67.99 |
|  | *Oribacterium sinus* | 0.257 | 0.358 | 2.506 | 2.768 | 70.76 |
|  | *Megasphaera micronuciformis* | 0.729 | 0.238 | 2.393 | 2.643 | 73.4 |
|  | ***Staphylococcus aureus*** | 0.0172 | 1.76 | 2.237 | 2.47 | 75.87 |
|  | *Actinomyces odontolyticus* | 0.282 | 0.247 | 1.832 | 2.023 | 77.89 |
|  | *Prevotella pallens* | 0.471 | 0.156 | 1.654 | 1.827 | 79.72 |
|  | *Prevotella nanceiensis* | 0.305 | 0.221 | 1.431 | 1.58 | 81.3 |
|  | *Parvimonas micros* | 0.333 | 0.685 | 1.307 | 1.443 | 82.74 |
|  | *Atopobium parvulum* | 0.0627 | 0.102 | 1.167 | 1.288 | 84.03 |
|  | *Porphyromonas endodontalis* | 0.416 | 0.072 | 1.108 | 1.223 | 85.26 |
|  | *Prevotella nigrescens* | 0.242 | 0.493 | 1.081 | 1.193 | 86.45 |
|  | *Prevotella denticola* | 0.0533 | 0.135 | 1.036 | 1.145 | 87.59 |
|  | *Capnocytophaga granulosa* | 0.17 | 0.0911 | 0.977 | 1.079 | 88.67 |
|  | *Lachnoanaerobaculum orale* | 0.218 | 0.122 | 0.9638 | 1.064 | 89.74 |
|  | *Prevotella loescheii* | 0.283 | 0.00487 | 0.8468 | 0.9352 | 90.67 |
